# Shorter-course treatment for *Mycobacterium ulcerans* disease with high-dose rifamycins and clofazimine in a mouse model of Buruli ulcer

**DOI:** 10.1371/journal.pntd.0006728

**Published:** 2018-08-13

**Authors:** Paul J. Converse, Deepak V. Almeida, Rokeya Tasneen, Vikram Saini, Sandeep Tyagi, Nicole C. Ammerman, Si-Yang Li, Nicole M. Anders, Michelle A. Rudek, Jacques H. Grosset, Eric L. Nuermberger

**Affiliations:** 1 Department of Medicine, Johns Hopkins University Center for Tuberculosis Research, Baltimore, Maryland, United States of America; 2 Analytical Pharmacology Core, The Sidney Kimmel Comprehensive Cancer Center, Johns Hopkins University School of Medicine, Baltimore, Maryland, United States of America; University of Tennessee, UNITED STATES

## Abstract

Starting in 2004, the standard regimen for treatment of Buruli ulcer (BU) recommended by the World Health Organization has been daily treatment for eight weeks with rifampin (RIF) and streptomycin. Based on recent clinical trials, treatment with an all-oral regimen of RIF and clarithromycin (CLR) may be an effective alternative. With the achievement of an all-oral regimen, a new goal is to find a regimen that can shorten the duration of treatment without compromising efficacy. We recently observed that increasing the dose of RIF from the standard 10 mg/kg dose to 20 or 40 mg/kg, or replacing RIF with the more potent long-acting rifamycin, rifapentine (RPT) at 10 mg/kg or 20 mg/kg increased the bactericidal activity of the RIF+CLR regimen in a mouse model of BU. We also recently showed that replacing CLR with clofazimine(CFZ) at 25 mg/kg may have greater sterilizing activity than the RIF+CLR regimen. Here, we demonstrate that combining high-dose rifamycins with CFZ at a lower dose of 12.5 mg/kg results in similar reductions in swelling, bacterial burden and mycolactone concentrations in mouse footpads compared to the standard regimens and more rapid sterilization of footpads as determined by the proportions of footpads harboring viable bacteria three months after completion of treatment. The potential of these high-dose rifamycin and CFZ combinations to shorten BU treatment to four weeks warrants evaluation in a clinical trial.

**Trial registration:**

ClinicalTrials.gov NCT03474198, NCT01659437

## Author summary

Buruli ulcer, a neglected tropical skin disease caused by *Mycobacterium ulcerans*, is treatable since 2004 with antibiotics instead of surgery. Treatment with either rifampin plus streptomycin or, more recently, rifampin plus clarithromycin requires taking the drugs daily for 8 weeks. Streptomycin is administered by injection and may result in hearing loss. Clarithromycin often causes gastrointestinal discomfort. Our goal is to identify a regimen that is both shorter and associated with fewer side effects. Rifampin, previously an expensive drug, is well tolerated not only at the standard dose of 10 mg/kg but at doses of 20 and 40 mg/kg. The related rifamycin, rifapentine, has a longer half-life and is also well tolerated. We tested in a mouse model of Buruli ulcer whether higher doses of these rifamycins together with clofazimine, a drug that has transient skin pigmentation side effects but no toxicities, could effectively reduce lesion size, the number of bacteria, and production of the mycolactone toxin, in a shorter time than that for the existing drug regimens. We found that treatment for 4 weeks with a high dose rifamycin plus clofazimine is as effective as 8 weeks of the current standard regimens of rifampin plus streptomycin or rifampin plus clarithromycin.

## Introduction

Buruli ulcer (BU), or *Mycobacterium ulcerans* disease, is a necrotizing skin disease driven by production of the immunosuppressive and cytotoxic macrolide-like toxin, mycolactone. Treatment for BU shifted from surgery and skin grafting to antibiotic therapy following pre-clinical and clinical evidence that combination, as opposed to single drug, therapy could be highly efficacious in killing *M*. *ulcerans*, stopping disease progression, reversing tissue damage and preventing relapse after treatment [1–3]. The original regimen of rifampin (RIF, 10 mg/kg) and streptomycin (STR, 15 mg/kg) given daily has both the benefit and the drawback of the inclusion of an injectable drug: patient and provider adherence is better assured but daily injections disrupt work, study, and recreation and burden the healthcare system. Although ototoxicity and other complications of STR treatment were not initially observed in West African patients based on self-report, more objective audiometric testing showed that hearing loss does indeed occur [4]. In Australia, which also has a large number of cases, all-oral antibiotic therapy combined with surgery has been in practice for many years [5]. In March of 2017, the BU Technical Advisory Group for the World Health Organization Global BU Initiative, due to difficulties in obtaining STR and preliminary findings of non-inferiority in a clinical trial (NCT01659437) in West Africa, recommended replacing STR with oral clarithromycin (CLR, 15–30 mg/kg) [6]. Compared to other chronic mycobacterial diseases such as leprosy and tuberculosis (TB) requiring antibiotic treatment for 6 to 24 months, depending on the form of the disease in each case, the treatment of BU with either regimen is relatively short at only 2 months. With the achievement of an all-oral regimen in hand, the next goal is to find ways to shorten treatment to one month or less.

Previous studies in our laboratory established that clofazimine (CFZ) at a dose of 25 mg/kg, when used in combination with RIF, is as effective as either standard regimen (*i*.*e*., RIF+STR or RIF+CLR) in reducing footpad swelling, production of mycolactone, and the *M*. *ulcerans* burden in a mouse footpad model of BU [7]. The regimen was comparable to RIF+STR and was significantly superior to RIF+CLR in preventing relapse after treatment for 6 weeks. Subsequently, research in our group with a mouse model of TB has shown that the dose of CFZ can be halved from 25 to 12.5 mg/kg with no loss of efficacy and a reduction in the transient skin discoloration associated with the drug [8, 9]. The MIC for CFZ against both *M*. *tuberculosis* and *M*. *ulcerans* is approximately 0.25–0.5 μg/ml [7]. Recent studies in our group have shown that increasing the dose of RIF and the long half-life rifamycin, rifapentine (RPT) shortens the treatment duration needed to prevent relapse in mouse models of TB [10, 11]. More recently, we found that replacing RIF with high-dose RIF or RPT resulted in greater bactericidal activity compared to RIF+CLR in a mouse footpad model of BU (T. Omansen et al., 2017, P1621, ECCMID, p. 191, WHO Meeting on BU, p. 128). Likewise, Chauffour et al. [12] showed that the regimen of RPT 10 mg/kg together with CLR achieved superior bactericidal effects and sterilizing efficacy at least comparable to that of RIF+STR in a mouse footpad model of BU.

One complication in the treatment of BU in humans is a paradoxical worsening of the clinical appearance of lesions [13]. Given that CFZ has both antimicrobial and anti-inflammatory properties and treats erythema nodosum reactions in leprosy, it has the additional potential advantage over STR and, possibly, CLR to reduce the risk of such paradoxical worsening.

Here, we evaluated the bactericidal and sterilizing efficacy of a rifamycin plus CFZ at the lower CFZ dose of 12.5 mg/kg and at escalating doses of RIF from 10 to 20 or 40 mg/kg and of RPT from 10 to 20 mg/kg. The results indicate that increasing the rifamycin dose can significantly shorten the treatment duration necessary to achieve culture negativity and prevent relapse when combined with CFZ, which may be a superior alternative to CLR or STR as a companion drug of the rifamycin.

## Methods

### Bacteria

*M*. *ulcerans* 1059 (Mu1059), originally obtained from a patient in Ghana, was generously provided by Dr. Pamela Small, University of Tennessee. Autoluminescent Mu1059 (Mu1059AL) was generated in our laboratory [14, 15]. These strains both produce mycolactone A/B, and this toxin kills macrophages and fibroblasts *in vitro* [16, 17]. The Mu1059AL strain was passaged in mouse footpads before use in these studies. The bacilli were harvested from footpads with grade 2 level swelling, i.e., swelling with inflammation[18].

### Ethics statement

All animal procedures were conducted according to relevant national and international guidelines. The study was conducted adhering to the Johns Hopkins University guidelines for animal husbandry and was approved by the Johns Hopkins Animal Care and Use Committee, #MO17M13. Johns Hopkins University is in compliance with the Animal Welfare Act regulations and Public Health Service Policy and also maintains accreditation of its program by the private Association for the Assessment and Accreditation of Laboratory Animal Care International.

### Antibiotics

RIF, CFZ, and STR were purchased from Sigma (St. Louis, MO). RPT and CLR were prepared from Priftin (Sanofi) and generic CLR (Aurobindo Pharma, Hyderabad, India, Dayton, NJ, USA) tablets, respectively, purchased at a pharmacy. Stock RIF and RPT (with brief sonication) suspensions were prepared every two weeks in distilled water; STR and CLR solutions were prepared weekly in water; and CFZ was suspended weekly in an 0.05% (w/v) agarose solution in distilled water. All drugs were given 5 days per week in 0.2 ml. RIF (10, 20, and 40 mg/kg), RPT (10 and 20 mg/kg), CFZ (25 mg/kg and 12.5 mg/kg), and CLR (100 mg/kg) were administered by gavage. STR (150 mg/kg) was administered by subcutaneous injection (S1 Table). Doses for CLR and STR were chosen based on mean plasma exposures (i.e., area under the concentration-time curve over 24 hours post-dose) compared to human doses.

### Infection and CFU analysis

BALB/c mice (N = 292), age 4–6 weeks (Charles River, Wilmington, MA), were inoculated in both hind footpads with approximately 4.42 log_10_ (2.65 x10^4^) CFU of Mu1059AL in 0.03 ml PBS, resulting in a mean (±S.D.) CFU count of 3.53±0.37 log_10_ *M*. *ulcerans* per footpad three days after infection. Treatment began 38 days after infection when footpad swelling increased to approximately grade 2 [18], and there were 5.31±0.28 log_10_ CFU/footpad. Treatment with RIF+STR, RIF+CLR, RIF+CFZ, RPT+CFZ and RIF or RPT alone was administered for up to 6 weeks for the combination regimens and up to 4 weeks for the monotherapy regimens. Footpads were harvested before treatment initiation (Day 0) and after 1, 2, and 4 weeks of treatment from mice (6 footpads from 3 mice) for CFU and relative light unit RLU counts to assess luminescence and mycolactone detection. For relapse determinations, 10 mice (20 footpads) were held without treatment for approximately 12 weeks after completing a 4- or 6-week combination regimen treatment (See the overall experiment scheme, including each regimen evaluated, in S1 Table). Mice were euthanized if they reached grade 3 swelling on a scale of 0–4, as described [18]. Footpad tissue was harvested, minced with fine scissors, suspended in 1.5 ml PBS, serially diluted, and plated on Middlebrook selective 7H11 plates (Becton-Dickinson, Sparks, MD). Plates were incubated at 32°C and colonies were counted after 8–12 weeks of incubation.

### Autoluminescence analysis

Autoluminescence was assessed using a Turner Designs (TD 20/20) luminometer in both intact footpads and in suspensions of minced footpads in PBS. Values in the latter tended to be approximately 5 times higher than those obtained in intact footpads and only the suspension values are reported here. The values are reported as relative light units (RLU).

### Analysis of mycolactone A/B concentrations

Samples of footpad tissue were stored in PBS at -20°C prior to mycolactone quantification. Mycolactone was extracted from 50 μl of tissue homogenate with 0.2 ml of acetonitrile containing 100 ng/ml of the internal standard, itraconazole. The standard curve and quality controls were prepared in blank mouse EDTA plasma. After centrifugation, the supernatant was transferred into an autosampler vial for LC-MS/MS analysis. Separation was achieved with a Thermo Betasil Phenyl (50 × 2.1 mm, 3 μm) column at 40°C with a gradient. Mobile phase A was water containing 0.1% formic acid and mobile phase B was acetonitrile containing 0.1% formic acid. The gradient started with mobile phase B was held at 20% for 0.5 minutes and increased to 100% over 0.5 minutes; 100% mobile phase B was held for 2 minutes and then returned back to 20% mobile phase B and allowed to equilibrate for 2 minutes. Total run time was 5 minutes with a flow rate of 0.3 ml/min. The column effluent was monitored using a Sciex triple quadrupole 4500 mass spectrometry detector (Sciex, Foster City, CA, USA) using electrospray ionization operating in positive mode. The spectrometer was programmed to monitor the following Multiple Reaction Monitoring transitions: 765.4 → 429.3 for mycolactone and 705.3 → 392.0 for itraconazole. Calibration curves for mycolactone were computed using the area ratio peak of the analysis to the internal standard using a quadratic equation with a 1/x^2^ weighting function over the range of 0.5 to 100 ng/ml.

### Rifamycin resistance analysis

DNA from each individual colony was extracted by boiling in 1X TE (Tris-EDTA, pH 8.0) buffer for 5 minutes; 5 μl of the supernatant was then used for PCR. Specific primers, forward primer MU_rpoF 5’ CGACGACATCGACCACTTC 3’ and reverse primer MU_rpoR 5’ CGACAGTGAACCGATCAGAC 3’, were used to amplify a 400 bp region encompassing the rifampin resistance-determining region. The PCR product was then sequenced to identify the presence of any mutation. Colonies from untreated control groups were used as a negative control.

### Statistical analysis

GraphPad Prism 6 was used to compare group means by student’s T test and analysis of variance and group proportions by Fisher’s exact test, and to determine Spearman’s correlation coefficients to evaluate RLU and CFU correlations.

## Results

### Infection and treatment initiation

**Fig 1:**
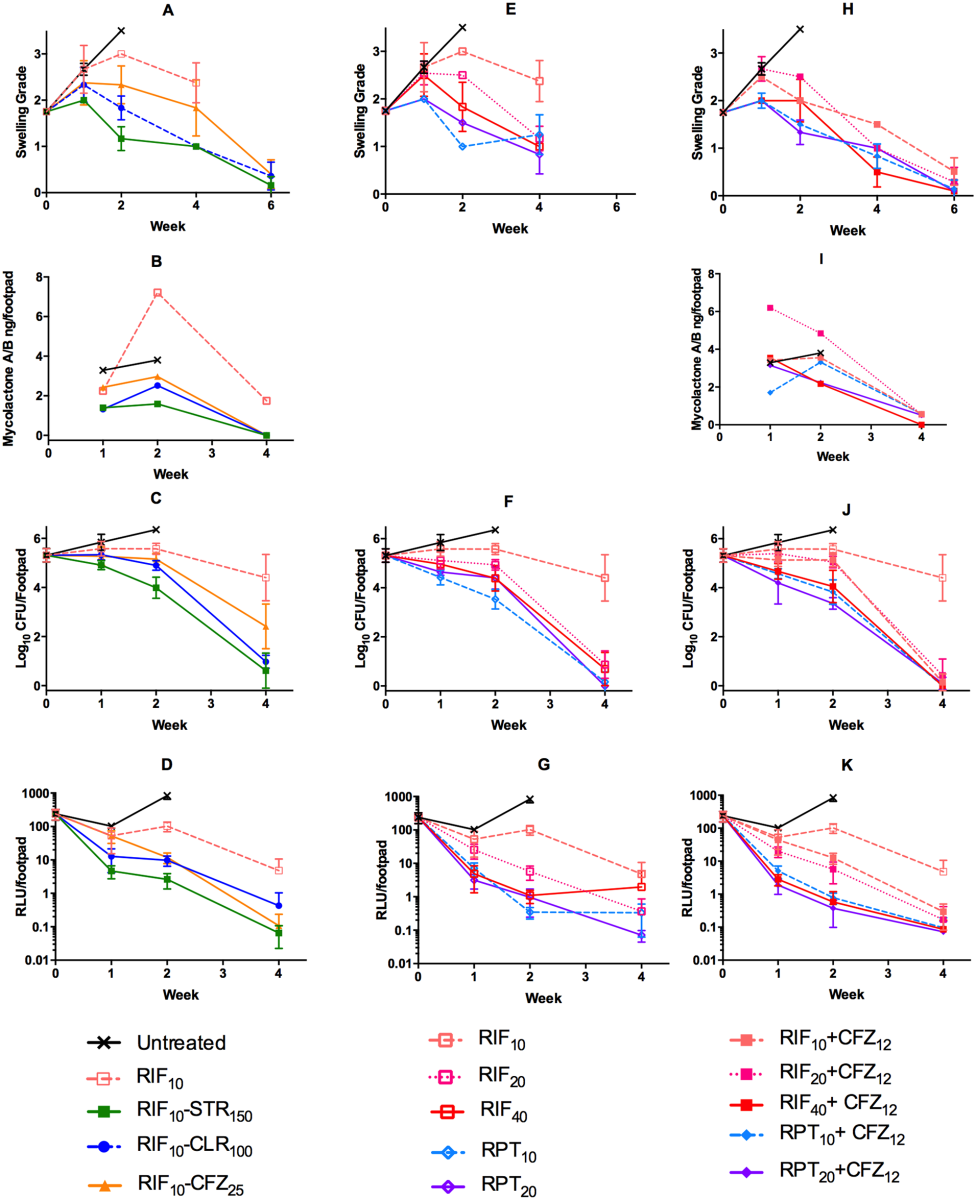
Response to treatment in *M*. *ulcerans*-infected mice treated with rifampin or rifapentine monotherapy at different doses or with standard-of-care regimens or regimens combining high-dose rifamycins with clofazimine. Mice were infected with *M*. *ulcerans* 1059AL. After 5 weeks the swelling grade was nearly 2 on a scale of 0–4 before treatment initiation (D0) with RIF monotherapy at the dose of 10 mg/kg (RIF_10_, open light red squares, dashed lines). Control mice were left untreated (x). Control combination regimens were RIF_10_ plus STR (150 mg/kg, green solid squares), CLR (100 mg/kg, blue solid circles), or CFZ (25 mg/kg, orange solid triangles). High-dose rifamycin monotherapy regimens included: RIF at 20 mg/kg (RIF_20_, open red square, dotted line) or 40 mg/kg (RIF_40_ open red square, solid line), or rifapentine at 10 mg/kg (RPT_10_, open light blue diamond, dashed line) or 20 mg/kg (RPT_20_, open purple diamond, solid line). Test combination regimens included CFZ (12.5 mg/kg) combined with RIF_10_ (solid light red square, dashed line), RIF_20_ (solid red square, dotted line), RIF_40_ (solid red square, solid line), RPT_10_ (solid light blue diamond, dashed line), or RPT_20_ (solid purple diamond, solid line). Mice were assessed over the next 4–6 weeks for footpad swelling (A, E, H), mycolactone concentration in footpads (B, I), bacterial burden in terms of colony forming units, CFU (C, F, J), or relative light units, RLU, in footpad homogenates (D, G, K).

Treatment was initiated five weeks after inoculation of *M*. *ulcerans* strain Mu1059AL, when the mean footpad swelling index was approximately 1.75 on a scale of 0–4 [21]. The mean CFU count on treatment initiation (Day 0) was 5.31 ± 0.28 log_10_, and the mean RLU count was 239.3 ± 84 (Fig 1).

### Response to treatment

#### RIF monotherapy and RIF-containing control regimens

Mice were evaluated daily by animal care staff and when mice were administered medication. At the initiation of treatment, swelling grade, a concurrent indicator of disease severity, was nearly identical in all the groups. Although swelling grade and CFU counts, obtained after >8 weeks of incubation, were not significantly different between untreated and RIF_10_ (i.e., RIF at 10 mg/kg) -treated mice after week 1, untreated mice experienced progressive footpad swelling and ulceration, which necessitated euthanasia at week 2. By this time, the swelling grade had advanced to ≥ 3.5 and 3.0 (p = 0.007) in untreated mice and mice receiving RIF_10_ alone, respectively (Fig 1A). Mycolactone A/B concentrations at week 2 were similar in untreated controls (mean, 5.78 ± 2.24 ng/footpad; median, 3.81) and in RIF_10_ alone-treated mice (mean, 8.26 ± 2.39 ng/footpad; median, 7.21) (Fig 1B). Mean CFU counts in footpads were 6.36 ± 0.05 and 5.58 ± 0.23 (p = 0.005) in control and RIF_10_ alone mice, respectively (Fig 1C). RLU values were 836.88 ± 186.8 and 102.6 ± 32.50 (p<0.0001) in untreated and RIF_10_ mice, respectively (Fig 1D). By week 4, only one mouse on RIF_10_ had been euthanized due to lesion worsening, and the reductions in footpad CFU and RLU counts, a real-time indicator of bacterial load, and mycolactone concentrations were all of similar magnitude. From these data, we conclude that treatment with RIF_10_ alone arrested disease progression but had a relatively modest impact on lesion size, mycolactone production and bacterial load over the first 4 weeks of treatment.

Among the control regimens, footpad swelling declined most rapidly with RIF_10_+STR, followed by RIF_10_+CLR and by RIF_10_+CFZ_25_ (Fig 1A). In comparison to untreated mice, swelling was significantly lower in the RIF_10_+STR group but not the other control groups at week 1. Mycolactone A/B concentrations increased in footpads of mice treated with any of the control regimens up to week 2, though to a lesser extent than in the RIF_10_ only group. RIF_10_+STR limited mycolactone production slightly more effectively than RIF_10_+CLR and RIF_10_+CFZ_25_. Mycolactone concentrations were reduced to near undetectable levels by week 4 with combination therapy but not with RIF_10_ alone (Fig 1B). As seen previously [7, 19], RIF_10_+STR was the most rapidly bactericidal control regimen. The difference with RIF_10_+CLR was statistically significant at weeks 1 and 2. As also found before, RIF_10_+CFZ_25_ was as effective as RIF_10_+CLR at week 2, but not week 4 (Fig 1C). RLU counts correlated well with CFU counts (e.g., Spearman *r*, >0.8 for RIF+STR) through week 2, before declining to near background levels, i.e., ≤1 RLU by week 4 (Fig 1D). Taken together, these results support the inclusion of any of the three non-rifamycin companion drugs in the regimen, with CFZ_25_ contributing similarly to CLR.

#### Rifamycin dose escalation (monotherapy)

While the combination controls were far more potent than RIF alone at the standard dose of 10 mg/kg, we also assessed the impact of increasing RIF and RPT doses as monotherapies. By week 1, a modest but detectable impact of increasing the rifamycin exposure was apparent with reductions in footpad swelling from a mean of 2.7 to 2.5 with increasing RIF doses while footpad swelling of all RPT-treated mice was 2.0. By week 2, the progression of swelling was arrested in RIF_20_-treated mice, while the swelling grade began to decline in RIF_40_-, RPT_10_- and RPT_20_-treated mice, reflecting a clear dose-dependent reduction in footpad swelling (Fig 1E). At week 4, mean swelling grades were similar for all high-dose rifamycin-treated mice and significantly lower than in RIF_10_-treated mice. CFU counts actually increased from Day 0 to week 2 in mice treated with RIF_10_ monotherapy, paralleling the swelling and RLU results, before declining modestly at Week 4. In contrast, there was a consistent and dose-dependent reduction in mice treated with RIF_20_ or RIF_40_ down to < 1 log_10_ per footpad. The difference between RIF_20_ and RIF_10_ was statistically significant (p = 0.02, p = 0.025, p<0.0001 at weeks 1, 2, and 4, respectively). In mice treated with RPT, there was an even greater reduction with mice having only 0.14 log_10_ (RPT_10_) or no cultivable bacteria (RPT_20_) at week 4, where CFU were numerically lower than in the RIF_10_+STR group (Fig 1F). RLU counts dropped more rapidly than CFU over the first 2 weeks of treatment but largely mirrored the CFU results in terms of ranking the regimens by efficacy (Fig 1G).

#### High-dose rifamycin regimens with clofazimine

Recent work in a murine TB model [8, 9] indicated that CFZ alone or in combination with first-line TB drugs has similar activity whether dosed at 12.5 mg/kg (CFZ_12.5_) or 25 mg/kg (CFZ_25_), while the former dose results in markedly less skin discoloration (S1 Fig, [8, 9]). Accordingly, in this experiment, mice were treated as before with RIF_10_+CFZ_25_ as a control and with RIF_10_+CFZ_12.5_ as well as with the higher doses of RIF, RIF_20_ or RIF_40_, or RPT_10_ or RPT_20_ with CFZ_12.5_. The RIF_10_+CFZ_12.5_ and RIF_20_+CFZ_12.5_ regimens produced a somewhat delayed reduction in footpad swelling relative to RIF_10_+STR and RIF_10_+CLR, consistent with the delayed activity of CFZ [8, 9], while RIF_40_+CFZ_12.5_ and the RPT+CFZ regimens caused reductions in swelling similar to the RIF_10_+STR and RIF_10_+CLR controls (Fig 1H). By 4 weeks, RIF_10_+CFZ_12.5_ and RIF_20_+CFZ_12.5_ were also similar to the control regimens. At week 2, the concentrations of mycolactone A/B were as high as or higher than the untreated controls (mean, 5.78±2.24; median, 3.81) in all of the test regimens except at the highest doses of RIF (RIF_40_+CFZ_12.5_: mean, 2.89±1.32; median 2.17) or RPT (RPT_20_+CFZ_12.5_: mean, 1.75±0.85; median, 2.23). At week 4, the concentrations in all combination regimen groups were <1 ng/footpad (Fig 1I). Likewise, with CFU counts, there was an initial stabilization and modest reduction compared to the increase in untreated mice and then a strong reduction between week 2 and week 4. Except for RIF_20_+CFZ_12.5_, all differences were highly statistically significant (p≤0.0005) compared to RIF_10_+CFZ_12.5_. RIF_40_-containing and both RPT-containing regimens were as effective as RIF_10_+STR over the first 2 weeks. By week 4, only one footpad of six was culture-positive in the RIF_10_+CFZ_12.5_ and RIF_20_+CFZ_12.5_ groups and none were culture-positive in the RIF_40_+CFZ_12.5_ group. In mice treated with RPT_10_+CFZ_12.5_ and RPT_20_+CFZ_12.5_, none was culture-positive in the former and only a single colony was found in the latter. In contrast, 3/6 and 6/6 were culture-positive in the RIF_10_+STR and RIF_10_+CLR groups, respectively (Fig 1J). RLU values declined more rapidly than CFU values as seen with the treatments reported above. RLU values in RPT-treated mice were reduced to near background levels by week 2 whereas in RIF-treated mice, background levels were only achieved by week 4 (Fig 1K).

#### Relapse results

All combination regimens were evaluated for their ability to prevent relapse after treatment for four or six weeks.

**Fig 2:**
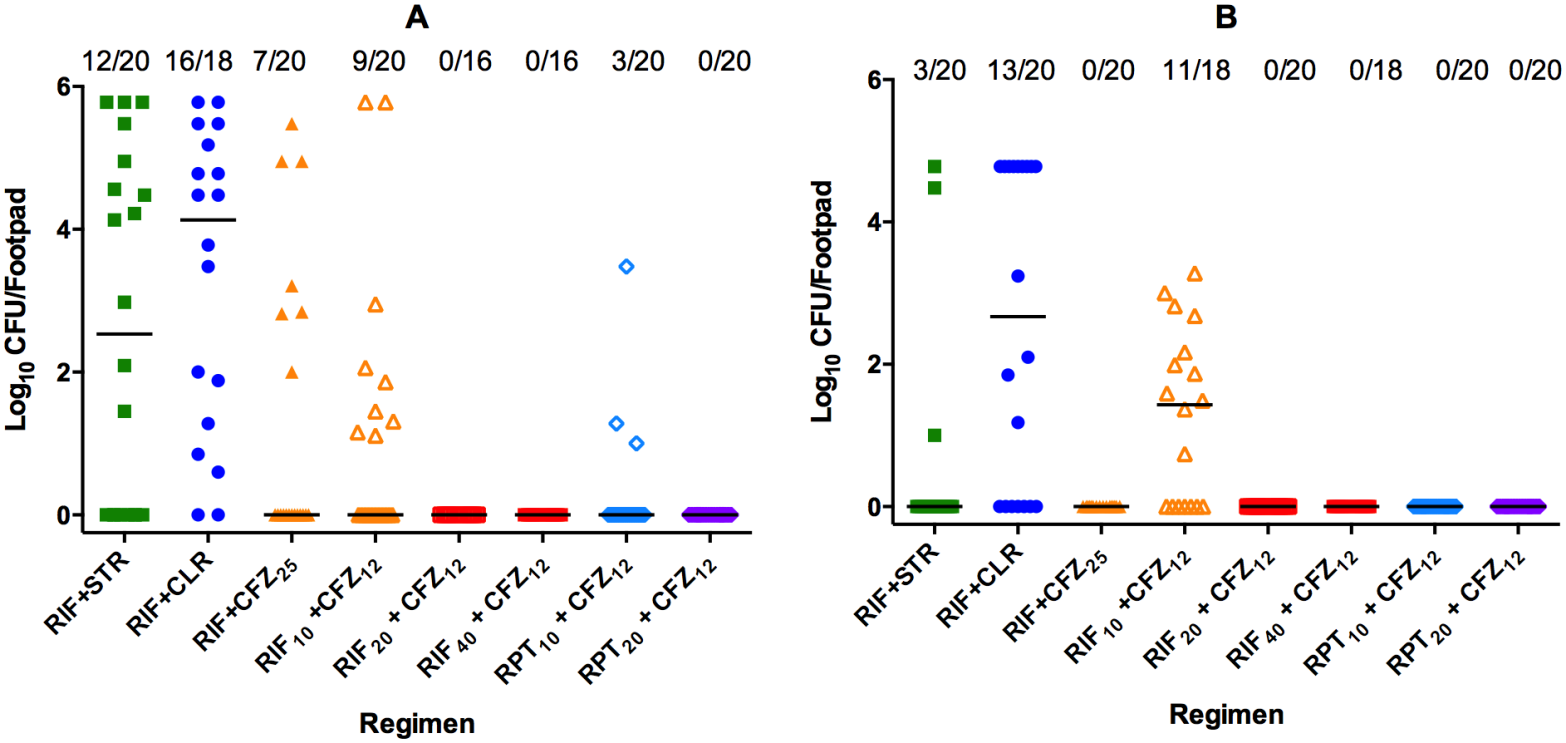
Relapse assessment in mice three months after completion of treatment with a combination regimen for four weeks (A) or six weeks (B). After treatment with one of the control or test combination regimens depicted in Fig 1, both footpads of up to 10 mice per group were assessed for bacterial burden. (RIF+STR, green solid squares), (RIF+CLR, solid blue circles), (RIF+CFZ_25_, solid orange triangles), (RIF_10_+CFZ_12_, open orange triangles), (RIF_20_+CFZ_12_, open red squares), (RIF_40_+CFZ_12_, solid red squares), (RPT_10_+CFZ_12_, light blue diamonds), (RPT_20_+CFZ_12_, purple diamonds). Relapse proportions are indicated above each treatment group. Because gavage accidents resulted in loss of 1 mouse in the RIF_10_+CFZ_25_, and 2 mice each in the RIF_20_+CFZ_12.5_ and RIF_40_+CFZ_12.5_ groups, these mice were lost to relapse evaluation.

Due to the long half-lives of both CFZ and RPT, it was hypothesized that these regimens might also be sterilizing after only 4 weeks of treatment. As expected, four weeks of treatment with the RIF_10_+STR or RIF_10_+CLR regimens failed to prevent relapses in most mice (Fig 2A). Relapses were observed in 12/20 (60%) and 16/18 (89%) footpads obtained mice treated with RIF_10_+STR and RIF_10_+CLR, respectively; this difference was not statistically significant. Treatment with standard-dose RIF, i.e., 10 mg/kg, plus CFZ at either 12.5 or 25 mg/kg was significantly better than RIF_10_+CLR (p = 0.02 and p<0.004, respectively) but relapse was observed in 9/20 (45%) in the RIF_10_+CFZ_12.5_ and 7/20 (35%) in the RIF_10_+CFZ_25_ footpads. The difference between the two CFZ doses was not statistically significant at this time point. Notably, treatment with higher RIF doses, i.e., either 20 or 40 mg/kg together with CFZ at 12.5 mg/kg prevented relapse in all 16 footpads assessed. Likewise, all 20 footpads of mice treated with RPT_20_+CFZ_12.5_ were culture-negative. Relapse was observed in three footpads from two mice treated with RPT_10_+CFZ_12.5_, but two of the footpads had low CFU numbers (Fig 2A). The proportion of relapsing footpads was significantly lower, even in the RPT_10_+CFZ_12.5_ group, compared to either RIF_10_+STR (p = 0.008) or RIF_10_+CLR (p<0.0001). In general, footpad swelling and RLU in footpad homogenates correlated with the CFU results (see Fig 1), particularly in the sterilizing regimens. In mice treated with RIF_10_+STR, RIF_10_+CLR, RIF_10_+CFZ_25_, and RIF_10_+CFZ_12.5_, mean RLU counts were 47.62, 46.24, 7.43, and 15.37, respectively, while in both high-dose RIF+CFZ_12.5_ and both RPT+CFZ_12.5_ groups, mean RLU counts were <1.

Mice were also evaluated for relapse 3 months after completing 6 weeks of treatment with the combination regimens. The additional two weeks of treatment reduced the proportion of relapses to 3/20 (15%) in RIF_10_+STR-treated mice and 13/20 (65%) in RIF_10_+CLR-treated mice (Fig 2B) (p = 0.003). The median CFU count was 0 for the RIF_10_+STR group but 2.57 log_10_ for the RIF_10_+CLR group. Mice treated with RIF_10_+CFZ_25_ were relapse-free while, among those treated with the same RIF dose but half the CFZ dose, 11/18 (61%) footpads relapsed with a median CFU count of 1.43 log_10_. Increasing the RIF dose to 20 or 40 mg/kg together with CFZ at 12.5 mg/kg rendered all mice relapse-free, indicating that, at this time point, either high-dose RIF or CFZ_25_ in combination with a low dose of the other drug was sufficient to sterilize footpads. Treatment with RPT at either 10 or 20 mg/kg prevented relapse in all mice. Footpad swelling was only observed in mice treated with RIF_10_+CLR (N = 10 footpads) and one footpad among mice treated with RIF_20_+CFZ_12.5_. Mean RLU values were <1.0 in footpads of mice treated with CFZ in any regimen while in RIF_10_+STR and RIF_10_+CLR-treated mice the means were 9.79 and 39.05, respectively.

#### Rifamycin resistance results

In order to determine if rifamycin resistance could account for the colonies found in 3 footpads in mice treated with RPT_10_+CFZ_12.5_, for 4 weeks and then assessed for relapse 3 months later (see Fig 2A), 3 colonies from each culture were subjected to DNA extraction followed by PCR of the *M*. *ulcerans rpoB* gene and sequencing. In all cases, the colonies were found to be identical to wild-type, i.e., with no mutations in *rpoB*.

## Discussion

Treatment for BU was revolutionized in the last 20 years after work in a mouse model like that employed here demonstrated that a combination of RIF_10_ plus either STR or amikacin could prevent the development of swelling and treat established lesions [18, 20, 21]. In humans, RIF+STR is efficacious but the inclusion of STR has the drawbacks of ototoxicity and a requirement for daily injections for 8 weeks [4]. In March, 2017, a WHO technical advisory group recommended the adoption of an all-oral regimen of RIF+CLR on the basis of a series of clinical trials [6, 22–24]. While better tolerated, the recommended regimen still requires 8 weeks of treatment. Therefore, shortening the duration of treatment required to safely eradicate *M*. *ulcerans* and its mycolactone toxin is now a major goal of research to improve BU treatment.

Increasing the rifamycin exposure by using higher doses of RIF or RPT increase the sterilizing activity of combination therapy in mouse models of TB [10, 11]. The treatment-shortening potential of anti-TB regimens containing daily RIF doses as high as 35 mg/kg [25] and RPT doses as high as 20 mg/kg [26] are now being evaluated in phase 2/3 trials (NCT02581527, NCT03474198, NCT02410772). We hypothesized that similar dose increases would shorten the duration of all-oral treatments for BU. Indeed, we found that, while monotherapy with the standard dose of RIF_10_ had a modest impact on swelling, mycolactone production, and bacterial burden, increasing the rifamycin dose had a significant impact on all three parameters, particularly on mycolactone levels and bacterial burden. Whereas RIF_20_ alone may have lagged behind the other high-dose rifamycins as monotherapy, they all had efficacy similar to that of the control regimens after 2–4 weeks of treatment. Combining high-dose RIF or RPT together with CFZ resulted in superior reduction of CFU burden and footpad swelling compared to rifamycin monotherapy, particularly after week 2. More importantly, these combinations significantly reduced the proportion of mice relapsing after 4 weeks of treatment compared to both RIF_10_+STR and RIF_10_+CLR. Combining CFZ with RIF_20_, RIF_40_ or RPT_20_ completely prevented relapse after 4 weeks of treatment, whereas RIF_10_+STR and RIF_10_+CLR treatment for 2 additional weeks was still associated with relapse in 15% and 65% of footpads, respectively. These results suggest that all-oral high-dose rifamycin and CFZ regimens have the potential to reduce the treatment duration from 8 weeks to 4 weeks without reducing efficacy. This would represent a substantial advance over the current standard of care.

CFZ was recently repurposed for treatment of multidrug-resistant TB as a component of a short-course regimen now endorsed by WHO [27]. There may be additional significant advantages to replacing CLR with CFZ in all-oral regimens for BU. RIF significantly induces human metabolism of CLR, lowering average CLR concentrations by 73–87% and compromising its activity as a companion agent [28, 29]. While CLR concentrations remain above the MIC when CLR is administered with standard doses of RIF to BU patients [30], the inductive effect of high-dose rifamycins is expected to be even greater and more consistent from person-to-person. The inductive effect of high-dose rifamycins is expected to be even greater and more consistent from person-to-person. CFZ, on the other hand, has no known unfavorable interactions with RIF. At the 100 mg human dose providing plasma exposures similar to the 12.5 mg/kg dose in mice, CFZ has better gastrointestinal tolerability than CLR. Given the anti-inflammatory activity of CFZ, treatment with this drug may reduce the possibility of paradoxical reactions [5, 13]. While the precise mechanisms underlying the induction of paradoxical reactions remain uncertain, accumulations of macrophages/giant cells have been observed. CFZ has been shown to accumulate in macrophages and to inhibit TNF production and boost anti-inflammatory IL-1RA production, including in dermal macrophages [31]. Giant cells form in response to both CD4-mediated and innate immune mechanisms [32]. Accordingly, we speculate that CFZ may modulate at least some of the inflammatory signals that may be involved in the induction of paradoxical reactions. Finally, CFZ is expected to become more readily available now due to its recommended use in the treatment of multi-drug resistant TB. Clinical trials are also underway (NCT03474198) or in the planning stages for combining rifamycins with CFZ in treatment of drug-susceptible TB based on treatment-shortening effects in a mouse model [8, 33, 34].

The main side effect of CFZ is skin discoloration. However, it should be emphasized that the discoloration is dose- and duration-dependent, is less noticeable in pigmented skin, and resolves completely after treatment completion [35–38]. We believe it is unlikely that 4 weeks of treatment producing plasma exposures observed with the 12.5 mg/kg dose used here in mice would produce noticeable or disconcerting skin discoloration. Any skin discoloration is expected to be a minor, short-lasting effect, particularly in comparison to the gastrointestinal intolerance associated with CLR [5, 39] and the toxicity and discomfort with STR [4]. CFZ may also be safer in BU patients with other co-morbidities. Caution in treating with CLR has been urged in patients with coronary heart disease in whom an increase in death has been observed after a two-week course of CLR. Deaths were apparent after patients were followed for one year or longer (NCT00121550) [40]. O’Brien *et al*. [5] noted that severe antibiotic complications developed at a median time of 4 weeks in Australian patients treated with the currently used oral regimens for BU, either RIF+CLR or RIF+fluoroquinolone, and were associated with reduced renal function.

Relapse of BU after antimicrobial treatment is thought to be rare, unlike in the preceding era when surgery without antibiotic treatment inevitably missed covert areas of infection. These observations suggest that there are new opportunities to shorten treatment durations with more potent drug combinations. To test the new regimens studied here, we used the stringent outcome measure of relapse prevention and found that high-dose rifamycins together with CFZ prevented relapse more effectively than RIF+STR and RIF+CLR despite shorter durations of treatment. Based on these promising results with drugs that are already in clinical use, these regimens warrant evaluation in clinical trials seeking to shorten the treatment of BU.

## Supporting information

S1 Fig Dose- and time- dependence of skin discoloration in mice treated with clofazimine (CFZ). 1) mice treated with rifampicin (RIF) and 25 mg/kg CFZ for 6 weeks; 2) mice treated with RIF and 12.5 mg/kg CFZ for 6 weeks; 3) mice treated with RIF and 25 mg/kg for 4 weeks and assessed two weeks later; and, 4) mice treated with rifampicin and streptomycin (STR) for 6 weeks. Discoloration, which likely occurs more rapidly in mice than in humans based on evidence from pharmacokinetic data, is most evident in the ears of mice treated with the highest dose (25 mg/kg) of CFZ but is much less in mice treated with half that dose. Mice treated with the highest dose for 4 weeks, followed by two weeks without treatment, resemble the mice treated with the lower dose for 6 weeks, illustrating the transient nature of drug-induced skin discoloration. Normal ear coloration is seen in mice treated with RIF+STR.
(PDF)

S1 Table Scheme of the experiment. (PDF)

S2 Table Footpad swelling grades. (PDF)

S3 Table Mycolactone values. (PDF)

S4 Table CFU data. (PDF)

S5 Table RLU data. (PDF)

## References

[1] Chauty A, Ardant M-F, Adeye A, Euverte H, Guedenon A, Johnson C, et al Promising clinical efficacy of streptomycin-rifampin combination for treatment of Buruli Ulcer (*Mycobacterium ulcerans* Disease). Antimicrob Agents Chemother. 2007;51(11):4029–35. 10.1128/AAC.00175-07 17526760 PMC2151409

[2] Converse PJ, Nuermberger EL, Almeida DV, Grosset JH. Treating *Mycobacterium ulcerans* disease (Buruli ulcer): from surgery to antibiotics, is the pill mightier than the knife? Future Microbiology. 2011;6(10):1185–98. 10.2217/fmb.11.101 22004037 PMC3243445

[3] Etuaful S, Carbonnelle B, Grosset J, Lucas S, Horsfield C, Phillips R, et al Efficacy of the Combination Rifampin-Streptomycin in Preventing Growth of *Mycobacterium ulcerans* in Early Lesions of Buruli Ulcer in Humans. Antimicrob Agents Chemother. 2005;49(8):3182–6. 10.1128/AAC.49.8.3182-3186.2005 16048922 PMC1196249

[4] Klis S, Stienstra Y, Phillips RO, Abass KM, Tuah W, van der Werf TS. Long Term Streptomycin Toxicity in the Treatment of Buruli Ulcer: Follow-up of Participants in the BURULICO Drug Trial. PLoS Negl Trop Dis. 2014;8(3):e2739 10.1371/journal.pntd.0002739 24625583 PMC3953024

[5] O’Brien DP, Friedman D, Hughes A, Walton A, Athan E. Antibiotic complications during the treatment of *Mycobacterium ulcerans* disease in Australian patients. Internal Medicine Journal. 2017;47(9):1011–9. 10.1111/imj.13511 28585259

[6] World Health Organization. Report from the Meeting of the Buruli ulcer Technical Advisory Group World Health Organization. Geneva, Switzerland: 2017 21 March 2017.

[7] Converse PJ, Tyagi S, Xing Y, Li SY, Kishi Y, Adamson J, et al Efficacy of Rifampin Plus Clofazimine in a Murine Model of *Mycobacterium ulcerans* Disease. PLoS Negl Trop Dis. 2015;9(6):e0003823 Epub 2015/06/05. 10.1371/journal.pntd.0003823 . 26042792 PMC4714850

[8] Ammerman NC, Swanson RV, Bautista EM, Almeida DV, Saini V, Omansen TF, et al Impact of Clofazimine Dosing on Treatment-Shortening of the First-Line Regimen in a Mouse Model of Tuberculosis. Antimicrob Agents Chemother. 2018;(1098–6596 (Electronic)). 10.1128/AAC.00636-18 PMC6021677 29735562

[9] Swanson RV, Adamson J, Moodley C, Ngcobo B, Ammerman NC, Dorasamy A, et al Pharmacokinetics and Pharmacodynamics of Clofazimine in a Mouse Model of Tuberculosis. Antimicrobial Agents and Chemotherapy. 2015;59(6):3042–51. 10.1128/AAC.00260-15 25753644 PMC4432183

[10] Rosenthal IM, Tasneen R, Peloquin CA, Zhang M, Almeida D, Mdluli KE, et al Dose-Ranging Comparison of Rifampin and Rifapentine in Two Pathologically Distinct Murine Models of Tuberculosis. Antimicrobial Agents and Chemotherapy. 2012;56(8):4331–40. 10.1128/AAC.00912-12 22664964 PMC3421552

[11] Rosenthal IM, Zhang M, Williams KN, Peloquin CA, Tyagi S, Vernon AA, et al Daily dosing of rifapentine cures tuberculosis in three months or less in the murine model. PLoS Med. 2007;4(12):e344 10.1371/journal.pmed.0040344 . 18092886 PMC2140085

[12] Chauffour A, Robert J, Veziris N, Aubry A, Jarlier V. Sterilizing Activity of Fully Oral Intermittent Regimens against *Mycobacterium ulcerans* Infection in Mice. PLOS Neglected Tropical Diseases. 2016;10(10):e0005066 10.1371/journal.pntd.0005066 27755552 PMC5068736

[13] O’Brien DP, Robson ME, Callan PP, McDonald AH. "Paradoxical" immune-mediated reactions to *Mycobacterium ulcerans* during antibiotic treatment: a result of treatment success, not failure. The Medical journal of Australia. 2009;191(10):564–6. Epub 2009/11/17. . 19912091 10.5694/j.1326-5377.2009.tb03313.x

[14] Zhang T, Li S-Y, Nuermberger EL. Autoluminescent *Mycobacterium tuberculosis* for Rapid, Real-Time, Non-Invasive Assessment of Drug and Vaccine Efficacy. PLoS ONE. 2012;7(1):e29774 10.1371/journal.pone.0029774 22253776 PMC3256174

[15] Zhang T, Li SY, Converse PJ, Grosset JH, Nuermberger EL. Rapid, Serial, Non-invasive Assessment of Drug Efficacy in Mice with Autoluminescent *Mycobacterium ulcerans* Infection. PLoS Negl Trop Dis. 2013;7(12):e2598 Epub 2013/12/25. 10.1371/journal.pntd.0002598 . 24367713 PMC3868507

[16] Converse PJ, Almeida DV, Nuermberger EL, Grosset JH. BCG-Mediated Protection against *Mycobacterium ulcerans* Infection in the Mouse. PLoS Neglected Tropical Diseases. 2011;5(3):e985 10.1371/journal.pntd.0000985 . 21423646 PMC3057947

[17] Zhang T, Li S-Y, Converse PJ, Almeida DV, Grosset JH, Nuermberger EL. Using Bioluminescence To Monitor Treatment Response in Real Time in Mice with *Mycobacterium ulcerans* Infection. Antimicrob Agents Chemother. 2011;55(1):56–61. 10.1128/AAC.01260-10 21078940 PMC3019670

[18] Dega H, Bentoucha A, Robert J, Jarlier V, Grosset J. Bactericidal activity of rifampin-amikacin against *Mycobacterium ulcerans* in mice. Antimicrob Agents Chemother. 2002;46(10):3193–6. 10.1128/AAC.46.10.3193-3196.2002 12234844 PMC128793

[19] Almeida D, Converse PJ, Ahmad Z, Dooley KE, Nuermberger EL, Grosset JH. Activities of Rifampin, Rifapentine and Clarithromycin Alone and in Combination against *Mycobacterium ulcerans* Disease in Mice. PLoS Negl Trop Dis. 2011;5(1):e933 10.1371/journal.pntd.0000933 21245920 PMC3014976

[20] Bentoucha A, Robert J, Dega H, Lounis N, Jarlier V, Grosset J. Activities of new macrolides and fluoroquinolones against *Mycobacterium ulcerans* infection in mice. Antimicrob Agents Chemother. 2001;45(11):3109–12. 10.1128/AAC.45.11.3109-3112.2001 . 11600364 PMC90790

[21] Dega H, Robert J, Bonnafous P, Jarlier V, Grosset J. Activities of several antimicrobials against *Mycobacterium ulcerans* infection in mice. Antimicrob Agents Chemother. 2000;44(9):2367–72. . 10952581 10.1128/aac.44.9.2367-2372.2000 PMC90071

[22] Chauty A, Ardant M-Fß, Marsollier L, Pluschke G, Landier J, Adeye A, et al Oral Treatment for *Mycobacterium ulcerans* Infection: Results From a Pilot Study in Benin. Clinical Infectious Diseases. 2011;52(1):94–6. 10.1093/cid/ciq072 21148526

[23] Nienhuis WA, Stienstra Y, Thompson WA, Awuah PC, Abass KM, Tuah W, et al Antimicrobial treatment for early, limited *Mycobacterium ulcerans* infection: a randomised controlled trial. The Lancet. 2010;375(9715):664–72. 10.1016/S0140-6736(09)61962-0 20137805

[24] Phillips RO, Sarfo FS, Abass MK, Abotsi J, Wilson T, Forson M, et al Clinical and Bacteriological Efficacy of Rifampin-Streptomycin Combination for Two Weeks followed by Rifampin and Clarithromycin for Six Weeks for Treatment of *Mycobacterium ulcerans* Disease. Antimicrobial Agents and Chemotherapy. 2014;58(2):1161–6. 10.1128/AAC.02165-13 24323473 PMC3910847

[25] Boeree MJ, Heinrich N, Aarnoutse R, Diacon AH, Dawson R, Rehal S, et al High-dose rifampicin, moxifloxacin, and SQ109 for treating tuberculosis: a multi-arm, multi-stage randomised controlled trial. The Lancet Infectious Diseases. 2017;17(1):39–49. 10.1016/S1473-3099(16)30274-2. 28100438 PMC5159618

[26] Dorman SE, Savic RM, Goldberg S, Stout JE, Schluger N, Muzanyi G, et al Daily rifapentine for treatment of pulmonary tuberculosis. A randomized, dose-ranging trial. Am J Respir Crit Care Med. 2015;191(3):333–43. Epub 2014/12/10. 10.1164/rccm.201410-1843OC . 25489785 PMC5447287

[27] Moodley R, Godec TR. Short-course treatment for multidrug-resistant tuberculosis: the STREAM trials. European Respiratory Review. 2016;25(139):29 10.1183/16000617.0080-2015 26929418 PMC9487666

[28] van Ingen J, Egelund EF, Levin A, Totten SE, Boeree MJ, Mouton JW, et al The pharmacokinetics and pharmacodynamics of pulmonary Mycobacterium avium complex disease treatment. Am J Respir Crit Care Med. 2012;186(6):559–65. Epub 2012/06/30. 10.1164/rccm.201204-0682OC . 22744719

[29] Wallace RJ Jr., Brown BA, Griffith DE, Girard W, Tanaka K. Reduced serum levels of clarithromycin in patients treated with multidrug regimens including rifampin or rifabutin for Mycobacterium avium-M. intracellulare infection. The Journal of infectious diseases. 1995;171(3):747–50. Epub 1995/03/01. 7876634 10.1093/infdis/171.3.747

[30] Alffenaar JW, Nienhuis WA, de Velde F, Zuur AT, Wessels AM, Almeida D, et al Pharmacokinetics of rifampicin and clarithromycin in patients treated for *Mycobacterium ulcerans* infection. Antimicrob Agents Chemother. 2010 Epub 2010/06/30. 10.1128/AAC.00099-10 . 20585115 PMC2935020

[31] Yoon GS, Keswani RK, Sud S, Rzeczycki PM, Murashov MD, Koehn TA, et al Clofazimine Biocrystal Accumulation in Macrophages Upregulates Interleukin 1 Receptor Antagonist Production To Induce a Systemic Anti-Inflammatory State. Antimicrobial Agents and Chemotherapy. 2016;60(6):3470–9. 10.1128/AAC.00265-16 27021320 PMC4879385

[32] Gharun K, Senges J, Seidl M, Lösslein A, Kolter J, Lohrmann F, et al Mycobacteria exploit nitric oxide-induced transformation of macrophages into permissive giant cells. EMBO reports. 2017;18(12):2144–59. 10.15252/embr.201744121 29097394 PMC5709734

[33] Grosset JH, Tyagi S, Almeida DV, Converse PJ, Li S-Y, Ammerman NC, et al Assessment of Clofazimine Activity in a Second-Line Regimen for Tuberculosis in Mice. American Journal of Respiratory and Critical Care Medicine. 2013;188(5):608–12. 10.1164/rccm.201304-0753OC 23822735 PMC3827279

[34] Tyagi S, Ammerman NC, Li S-Y, Adamson J, Converse PJ, Swanson RV, et al Clofazimine shortens the duration of the first-line treatment regimen for experimental chemotherapy of tuberculosis. Proceedings of the National Academy of Sciences. 2015;112(3):869–74. 10.1073/pnas.1416951112 25561537 PMC4311815

[35] Browne SG, Harman DJ, Waudby H, McDougall AC. Clofazimine (Lamprene, B663) in the treatment of lepromatous leprosy in the United Kingdom. A 12 year review of 31 cases, 1966–1978. International journal of leprosy and other mycobacterial diseases: official organ of the International Leprosy Association. 1981;49(2):167–76. Epub 1981/06/01. . 7196886

[36] Hastings RC, Jacobson RR, Trautman JR. Long-term clinical toxicity studies with clofazimine (B663) in leprosy. International journal of leprosy and other mycobacterial diseases: official organ of the International Leprosy Association. 1976;44(3):287–93. Epub 1976/07/01. . 824210

[37] U.S. Leprosy Panel, Leonard Wood Memorial. Spaced clofazimine therapy of lepromatous leprosy. The American journal of tropical medicine and hygiene. 1976;25(3):437–44. Epub 1976/05/01. . 779502 10.4269/ajtmh.1976.25.437

[38] Yawalkar SJ, Vischer W. Lamprene (clofazimine) in leprosy. Basic information. Leprosy review. 1979;50(2):135–44. Epub 1979/06/01. . 396428 10.5935/0305-7518.19790020

[39] Williams KN, Bishai WR. Clarithromycin extended-release in community-acquired respiratory tract infections. Expert Opin Pharmacother. 2005;6(16):2867–76. 10.1517/14656566.6.16.2867 . 16318437

[40] U.S. Food and Drug Administration. Clarithromycin (Biaxin): Drug Safety Communication—Potential Increased Risk of Heart Problems or Death in Patients With Heart Disease. In: Services DoHaH, editor. 2018.

