# Supplementary material for: Shorter-course treatment for Mycobacterium ulcerans disease with high-dose rifamycins and clofazimine in a mouse model of Buruli ulcer
Source: PLoS Negl Trop Dis. 2018 Aug 13;12(8):e0006728. doi: 10.1371/journal.pntd.0006728 (PMC6107292; doi:10.1371/journal.pntd.0006728)
Supplement: S1 Fig — 1) mice treated with rifampicin (RIF) and 25 mg/kg CFZ for 6 weeks; 2) mice treated with RIF and 12.5 mg/kg CFZ for 6 weeks; 3) mice treated with RIF and 25 mg/kg for 4 weeks and assessed two weeks later; and, 4) mice treated with rifampicin and streptomycin (STR) for 6 weeks. Discoloration, which likely occurs more rapidly in mice than in humans based on evidence from pharmacokinetic data, is most evident in the ears of mice treated with the highest dose (25 mg/kg) of CFZ but is much less in mice treated with half that dose. Mice treated with the highest dose for 4 weeks, followed by two weeks without treatment, resemble the mice treated with the lower dose for 6 weeks, illustrating the transient nature of drug-induced skin discoloration. Normal ear coloration is seen in mice treated with RIF+STR. (PDF) [file pntd.0006728.s001.pdf]

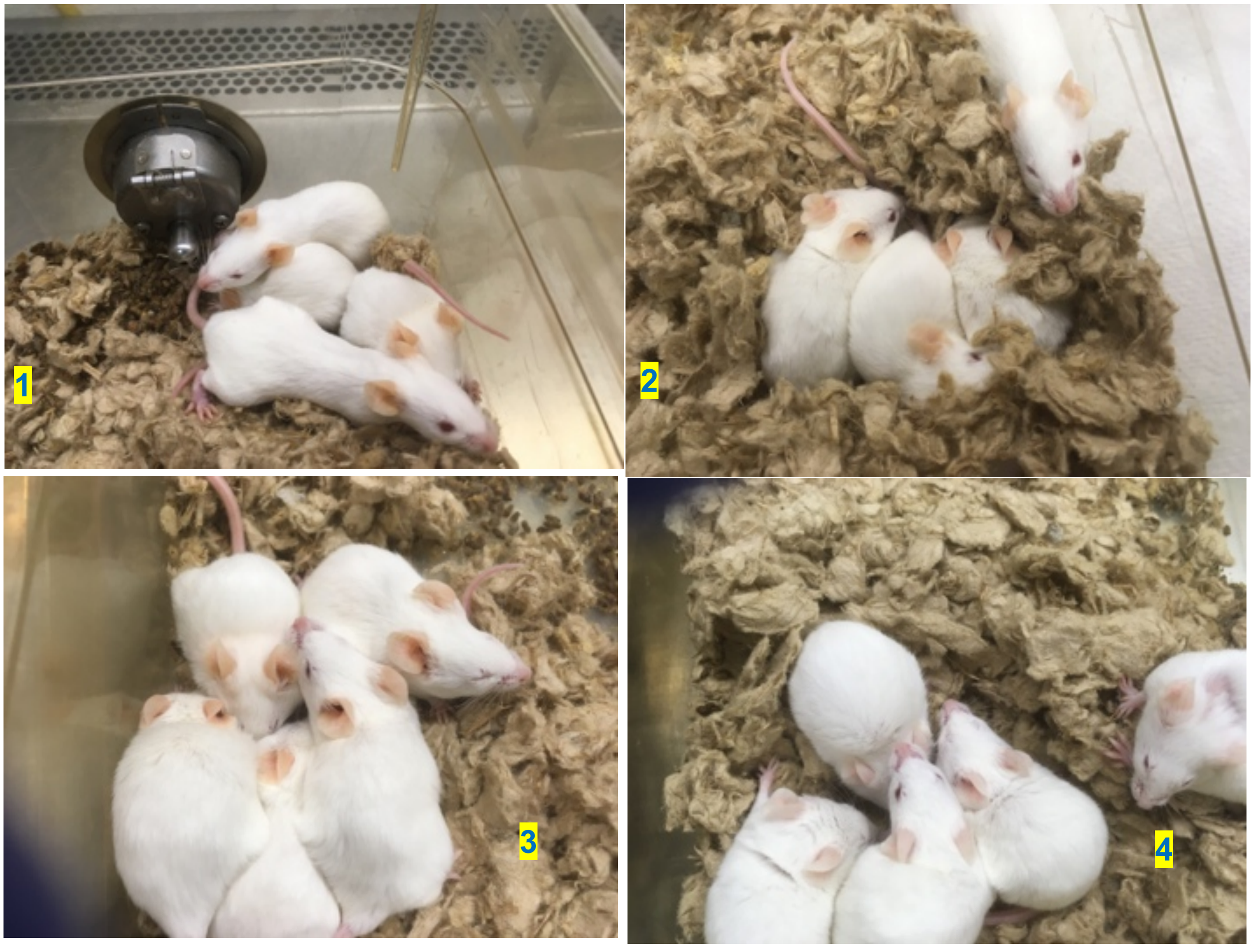

S1 Fig.

Dose- and time- dependence of skin discoloration in mice treated with clofazimine (CFZ).

1) mice treated with rifampicin (RIF) and 25 mg/kg CFZ for 6 weeks; 2) mice treated with RIF and 12.5 mg/kg CFZ for 6 weeks; 3) mice treated with RIF and 25 mg/kg for 4 weeks and assessed two weeks later; and, 4) mice treated with rifampicin and streptomycin (STR) for 6 weeks.

Discoloration, which likely occurs more rapidly in mice than in humans based on evidence from pharmacokinetic data, is most evident in the ears of mice treated with the highest dose (25 mg/kg) of CFZ but is much less in mice treated with half that dose. Mice treated with the highest dose for 4 weeks, followed by two weeks without treatment, resemble the mice treated with the lower dose for 6 weeks, illustrating the transient nature of drug-induced skin discoloration. Normal ear coloration is seen in mice treated with RIF+STR.
