# Supplementary material for: Shorter-course treatment for Mycobacterium ulcerans disease with high-dose rifamycins and clofazimine in a mouse model of Buruli ulcer
Source: PLoS Negl Trop Dis. 2018 Aug 13;12(8):e0006728. doi: 10.1371/journal.pntd.0006728 (PMC6107292; doi:10.1371/journal.pntd.0006728)
Supplement: S1 Table — (PDF) [file pntd.0006728.s002.pdf]

Supporting Information

S1 Table. Scheme of the Experiment

**Treatment group**	**Time point and number of mice sacrificed for footpad CFU counts**							
**Controls**	D-35	D0	W1	W2	W4	W4+12	W6+12	**Total**
Untreated	3	3	3	3	3			**15**
RIF_{10}+STR_{150}			3	3	3	10	10	**29**
RIF_{10}+CLR_{100}			3	3	3	10	10	**29**
RIF_{10}+CFZ_{25}			3	3	3	10	10	**29**
RIF_{10}			3	3	3			**9**
**Rifamycin Tests**								
RIF_{20}			3	3	3			**9**
RIF_{40}			3	3	3			**9**
RPT_{10}			3	3	3			**9**
RPT_{20}			3	3	3			**9**
**Tests with CFZ**								
RIF_{10}+$CFZ_{12.5}$			3	3	3	10	10	**29**
RIF_{20}+$CFZ_{12.5}$			3	3	3	10	10	**29**
RIF_{40}+$CFZ_{12.5}$			3	3	3	10	10	**29**
RPT_{10}+$CFZ_{12.5}$			3	3	3	10	10	**29**
RPT_{20}+$CFZ_{12.5}$			3	3	3	10	10	**29**
**Total**	**3**	**3**	**42**	**42**	**42**	**80**	**80**	**292**

RIF, rifampin; STR, streptomycin; CLR, clarithromycin; CFZ, clofazimine; RPT, rifapentine. Doses (in mg/kg) for each drug are given in the subscript.
