# Supplementary material for: Shorter-course treatment for Mycobacterium ulcerans disease with high-dose rifamycins and clofazimine in a mouse model of Buruli ulcer
Source: PLoS Negl Trop Dis. 2018 Aug 13;12(8):e0006728. doi: 10.1371/journal.pntd.0006728 (PMC6107292; doi:10.1371/journal.pntd.0006728)
Supplement: S2 Table — (PDF) [file pntd.0006728.s003.pdf]

WEEK 1 Swelling

	1L	2L	3L	1R	2R	3R
	**FP Swell**	**FP Swell**	**FP Swell**	**FP Swell**	**FP Swell**	**FP Swell**
UT	2.5	2.75	2.75	2.5	2.75	2.75
RS	2	2	2	2	2	2
RCLR	3	2	2.5	2.25	2	2.25
R10C25	2	2.5		2	3	
R10	2	3	3	2	3	3
R20	2.5	2.5	3	2.25	2.5	2.5
R40	2.5	3	2	2.5	3	2
P10	2	2	2	2	2	2
P20	2	2	2	2	2	2
R10C12	2.5	2.5	2.5	2.5	2.5	2.5
R20C12	2.5	2.5	3	2.5	2.5	3
R40C12	2	2	2	2	2	2
P10C12	2	2	2.25	2	2	1.75
P20C12	2	2	2	2	2	2

WEEK 2 Swelling

	1L	2L	3L	1R	2R	3R
	**FP Swell**	**FP Swell**	**FP Swell**	**FP Swell**	**FP Swell**	**FP Swell**
UT	3.5	3.5	3.5	3.5	3.5	3.5
RS	1	1	1.5	1	1	1.5
RCLR	1.5	2	2	1.5	2	2
R10C25	2.5	2.5	1.5	2.5	2.5	2.5
R10	3	3	3	3	3	3
R20	2.5	2.5	2.5	2.5	2.5	2.5
R40	1.5	1.5	2.5	1.5	1.5	2.5
P10	1	1	1	1	1	1
P20	1.5	1.5	1.5	1.5	1.5	1.5
R10C12	2	2	2	2	2	2
R20C12	2.5	2.5	2.5	2.5	2.5	2.5
R40C12	1.5	2	2.5	1.5	2	2.5
P10C12	1.5	1.5	1.5	1.5	1.5	1.5
P20C12	1.5	1.5	1	1.5	1.5	1

WEEK 4 Swelling

	1L	2L	3L	1R	2R	3R
	**FP Swell**	**FP Swell**	**FP Swell**	**FP Swell**	**FP Swell**	**FP Swell**
UT	--------	--------	--------	--------	--------	--------
RS	1	1	1	1	1	1
RCLR	1	1	1	1	1	1
R10C25	1.5	2.5	1	2	2.5	1.5
R10	3	2.25		2	2.25	
R20	1	1	1.5	1	1	1.5
R40	1	1	1	1	1	1
P10	1	1	1	1.5	1	2
P20	0.5	1	0.5	0.5	1	1.5
R10C12	1.5	1.5	1.5	1.5	1.5	1.5
R20C12	1	1	1	1	1	1
R40C12	0.5	0.5	0	0.5	1	0.5
P10C12	1	1	0.5	1	1	0.5
P20C12	1	1	1	1	1	1

Week 6, cages A and B, Footpads L and R

Regimen	Wk 6 A (L)	Wk 6 A (R)	Wk 6 B (L)	Wk 6 B (R)
RS	0.25, 0.25, 0.25, 0.25, 0.25,	0.25,,0,,0,0,0	0.5,0,0.5,0.25,0	0.5,0,0,0,0
RCLR	0.5,0.5,0.75,1,0.5	0,0,0,0.25,0.25	0.5,0.75,0.75,0.5,0.25	0,0.25,0.25,0.25,0
R10C25	0.75,0.75,0.5,0.75	0,0.25,0,0.25	0.5,0.75,0.5,0.5	0.25,0,0.25,0.25
R10C12	0.75,1,0.5,1,0.5	0.75,0.5,0,0.25,0.5	0.75,0.5,0.25,0.75	0.5,0.25,0,0.5
R20C12	0.25,0.75,0.75,0.25,0	0,0.5,0.5,0,0	1,0.25,0.5,0.25,0.25	0.5,0,0,0,0
R40C12	0.25,0.25,0.25,0.25,0.25	0,0,0,0,0	0.5,0,0,0.25	0,0,0,0
P10C12	0,0,0,0.25,0.5	0,0,0,0,0	0.5,0,0.5,0.25,0.25	0.25,0,0.25,0,0
P20C12	0.25,0.25,0.25,0.5,0	0,0,0,0,0	0.25,0.25,0,0.25,0	0,0,0,0,0
