# Supplementary material for: Shorter-course treatment for Mycobacterium ulcerans disease with high-dose rifamycins and clofazimine in a mouse model of Buruli ulcer
Source: PLoS Negl Trop Dis. 2018 Aug 13;12(8):e0006728. doi: 10.1371/journal.pntd.0006728 (PMC6107292; doi:10.1371/journal.pntd.0006728)
Supplement: S3 Table — (PDF) [file pntd.0006728.s004.pdf]

	A	B	C	D	E	F	G	H	I	J	K
1	WEEK	**UT**							**MEAN**	**SD**	**MEDIAN**
2	1	2.9	3.2	3.18	4.55	10.2	3.38		4.57	2.82	3.29
3	2	6.16	12.6	12.2	0.886	1.38	1.45		5.78	5.48	3.81
4	4										
5		**RS**							**RS**		
6	1	1.4	0	1.88					1.09	0.98	1.40
7	2	1.51	1.59	1.65					1.58	0.07	1.59
8	4	0	0	1.31					0.44	0.76	0.00
9		**RCLR**							**RCLR**		
10	1	0	1.67	1.32					1.00	0.88	1.32
11	2	3.54	2.52	2.26					2.77	0.68	2.52
12	4	0	0	0					0.00	0.00	0.00
13		**RCFZ**							**RCFZ**		
14	1	2.43	2.64	2.07					2.38	0.29	2.43
15	2	2.85	2.97	3.68					3.17	0.45	2.97
16	4	0	0	1.14					0.38	0.66	0.00
17		**RIF**							**RIF**		
18	1	2.25	2.32	1.89					2.15	0.23	2.25
19	2	7.21	11	6.58					8.26	2.39	7.21
20	4	2.02	1.58	1.75					1.78	0.22	1.75
21		**R10CFZ**							**R10CFZ**		
22	1	2.71	3.42	4.55					3.56	0.93	3.42
23	2	4.97	2.09	3.56					3.54	1.44	3.56
24	4	0.404	0.987	0.573					0.65	0.30	0.57
25		**R20CFZ**							**R20CFZ**		
26	1	6.2	2.26	6.33					4.93	2.31	6.20
27	2	7.68	4.84	2.67					5.06	2.51	4.84
28	4	0.549	0.831	0.538					0.64	0.17	0.55
29		**R40CFZ**							**R40CFZ**		
30	1	3.17	3.55	5.37					4.03	1.18	3.55
31	2	2.17	2.08	4.41					2.89	1.32	2.17
32	4	0	0	0					0.00	0.00	0.00
33		**P10CFZ**							**P10CFZ**		
34	1	1.7	0.892	2.24					1.61	0.68	1.70
35	2	4.91	2.06	3.32					3.43	1.43	3.32
36	4	0.908	0.574	0.401					0.63	0.26	0.57
37		**P20CFZ**							**P20CFZ**		
38	1	2.91	3.15	6.86					4.31	2.21	3.15
39	2	0.767	2.23	2.26					1.75	0.85	2.23
40	4	0.468	0.51	0.607					0.53	0.07	0.51
