# Supplementary material for: Shorter-course treatment for Mycobacterium ulcerans disease with high-dose rifamycins and clofazimine in a mouse model of Buruli ulcer
Source: PLoS Negl Trop Dis. 2018 Aug 13;12(8):e0006728. doi: 10.1371/journal.pntd.0006728 (PMC6107292; doi:10.1371/journal.pntd.0006728)
Supplement: S4 Table — (PDF) [file pntd.0006728.s005.pdf]

	A	B	C	D	E	F	G	H	I	J	K	L	M	N	O
1	**Inoculum**	0	6	44	340	2000	∞		44	20000	880001	5.94			
2	**Implantation**														
3	Footpads	**all plain**							avg cfu ct	dilution factor	(X+1)	log cfu	**mean log cfu**	**st dev**	
4			**-5**	**-4**	**-3**	**-2**	**-1**	**neat**							
5	Mouse 1L					18	150	1000	18	200	3601	3.56	**3.53**	**0.37**	
6	Mouse 1R					2	32	300	32	20	641	2.81			
7	Mouse 2L					25	250	2000	25	200	5001	3.70			
8	Mouse 2R					24	220	2000	24	200	4801	3.68			
9	Mouse 3L					35	200	∞	35	200	7001	3.85			
10	Mouse 3R					19	340	∞	19	200	3801	3.58			
11															
12	**Day 0**														
13	Footpads	**all plain**							avg cfu ct	dilution factor	(X+1)	log cfu	**mean log cfu**	**st dev**	
14			**-5**	**-4**	**-3**	**-2**	**-1**	**neat**							
15	Mouse 1L			9	90	300	∞		90	2000	180001	5.26	**5.31**	**0.28**	
16	Mouse 1R			6	68	300	∞		68	2000	136001	5.13			
17	Mouse 2L			1	57	300	∞		57	2000	114001	5.06			
18	Mouse 2R			8	68	300	∞		68	2000	136001	5.13			
19	Mouse 3L			29	220	300	∞		29	20000	580001	5.76			
20	Mouse 3R			17	150	300	∞		17	20000	340001	5.53			
21															

Week 1 Untreated

Footpads	all plain					avg cfu ct	dilution facto	(X+1)	log cfu	mean log cfu	st dev
	-4	-3	-2	-1	neat						
Mouse 1L	14	70	620	∞		14	20000	280001	5.45	**5.85**	**0.33**
Mouse 1R	39	330	∞	∞+		39	20000	780001	5.89		
Mouse 2L	69	500	∞	∞+		69	20000	1380001	6.14		
Mouse 2R	93	750	∞	∞+		93	20000	1860001	6.27		
Mouse 3L	33	150	∞	∞+		33	20000	660001	5.82		
Mouse 3R	16	64	700	∞		16	20000	320001	5.51		

Week 1 RIF-STR

Footpads	all plain					avg cfu ct	dilution facto	(X+1)	log cfu	mean log cfu	st dev
	-4	-3	-2	-1	neat						
Mouse 1L		23	70	550	∞	23	2000	46001	4.66	**4.91**	**0.18**
Mouse 1R		29	200	1000	∞	29	2000	58001	4.76		
Mouse 2L		72	300	∞	∞+	72	2000	144001	5.16		
Mouse 2R		45	300	∞	∞+	45	2000	90001	4.95		
Mouse 3L		37	190	1500	∞	37	2000	74001	4.87		
Mouse 3R		57	390	∞	∞+	57	2000	114001	5.06		

Week 1 RIF-CLR

Footpads	all plain					avg cfu ct	dilution facto	(X+1)	log cfu	mean log cfu	st dev
	-4	-3	-2	-1	neat						
Mouse 1L	19	100	1000	∞	∞+	19	20000	380001	5.58	**5.34**	**0.21**
Mouse 1R	5	94	600	∞	∞+	94	2000	188001	5.27		
Mouse 2L	2	45	200	∞	∞+	45	2000	90001	4.95		
Mouse 2R	13	100	1000	∞	∞+	13	20000	260001	5.41		
Mouse 3L	13	74	470	∞	∞+	13	20000	260001	5.41		
Mouse 3R	13	70	300	∞	∞+	13	20000	260001	5.41		

Week 1 RIF$_{10}$-CFZ$_{25}$

Footpads	all plain					avg cfu ct	dilution facto	(X+1)	log cfu	mean log cfu	st dev
	-4	-3	-2	-1	neat						
Mouse 1L	6	27	200	2000	∞+	27	2000	54001	4.73	**5.28**	**0.37**
Mouse 1R	5	59	350	∞	∞+	59	2000	118001	5.07		
Mouse 2L	6	102	700	∞	∞+	102	2000	204001	5.31		
Mouse 2R	23	180	800	∞		23	20000	460001	5.66		
Mouse 3L	6	73	360	∞	∞+	73	2000	146001	5.16		
Mouse 3R	26	200	1000	∞	∞+	26	20000	520001	5.72		

Week 1 RIF$_{10}$

Footpads	all plain					avg cfu ct	dilution facto	(X+1)	log cfu	mean log cfu	st dev

		-4	-3	-2	-1	neat						mean log cfu	st dev
Mouse 1L		34	120	1000	∞	∞+	34	20000	680001	5.83		**5.58**	**0.30**
Mouse 1R		19	79	750	∞	∞+	19	20000	380001	5.58			
Mouse 2L		19	60	600	∞	∞+	19	20000	380001	5.58			
Mouse 2R		46	200	∞	∞+	∞+	46	20000	920001	5.96			
Mouse 3L		2	66	250	∞	∞+	66	2000	132001	5.12			
Mouse 3R		13	120	760	∞	∞+	13	20000	260001	5.41			

Week 1 RIF$_{20}$

Footpads	**all plain**						avg cfu ct	dilution factor	(X+1)	log cfu		**mean log cfu**	**st dev**
		**-4**	**-3**	**-2**	**-1**	**neat**							
Mouse 1L		9	43	270	∞	∞+	43	2000	86001	4.93		**5.12**	**0.35**
Mouse 1R		5	41	200	∞	∞+	41	2000	82001	4.91			
Mouse 2L		27	200	900	∞	∞+	27	20000	540001	5.73			
Mouse 2R		2	46	200	∞	∞+	46	2000	92001	4.96			
Mouse 3L		4	33	150	1100	∞+	33	2000	66001	4.82			
Mouse 3R		11	110	700	∞	∞+	11	20000	220001	5.34			

Week 1 RIF$_{40}$

Footpads	**all plain**						avg cfu ct	dilution factor	(X+1)	log cfu		**mean log cfu**	**st dev**
		**-4**	**-3**	**-2**	**-1**	**neat**							
Mouse 1L		5	32	170	∞	∞+	32	2000	64001	4.81		**4.96**	**0.11**
Mouse 1R		7	65	300	∞	∞+	65	2000	130001	5.11			
Mouse 2L		3	51	200	∞	∞+	51	2000	102001	5.01			
Mouse 2R		5	51	170	∞	∞+	51	2000	102001	5.01			
Mouse 3L		4	48	160	∞	∞+	48	2000	96001	4.98			
Mouse 3R		**21**	**26**	350	∞	∞+	350	200	70001	4.85			
		dilution error	mixed -3 and -4, use -2										

Week 1 RPT$_{10}$

Footpads	**all plain**						avg cfu ct	dilution factor	(X+1)	log cfu		**mean log cfu**	**st dev**
		**-4**	**-3**	**-2**	**-1**	**neat**							
Mouse 1L		1	4	55	500	∞	55	200	11001	4.04		**4.42**	**0.30**
Mouse 1R		4	30	200	1000	∞	30	2000	60001	4.78			
Mouse 2L		3	7	87	800	∞	87	200	17401	4.24			
Mouse 2R		5	9	85	500	∞	85	200	17001	4.23			
Mouse 3L		1	15	150	900	∞	150	200	30001	4.48			
Mouse 3R		0	29	200	1100	∞	29	2000	58001	4.76			

Week 1 RPT$_{20}$

Footpads	**all plain**						avg cfu ct	dilution factor	(X+1)	log cfu		**mean log cfu**	**st dev**
		**-4**	**-3**	**-2**	**-1**	**neat**							
Mouse 1L		0	20	175	850	∞	20	2000	40001	4.60		**4.64**	**0.13**

Mouse 1R		0	31	200	1200	∞	31	2000	62001	4.79			
Mouse 2L		4	32	200	1200	∞	32	2000	64001	4.81			
Mouse 2R		4	15	150	900	∞	15	2000	30001	4.48			
Mouse 3L		4	17	150	850	∞	17	2000	34001	4.53			
Mouse 3R		0	22	170	∞	∞+	22	2000	44001	4.64			

Week 1 RIF$_{10}$-CFZ$_{12.5}$

Footpads	**all plain**						avg cfu ct	dilution facto	(X+1)	log cfu		**mean log cfu**	**st dev**
		**-4**	**-3**	**-2**	**-1**	**neat**							
Mouse 1L		3	40	250	∞	∞	40	2000	80001	4.90		**5.13**	**0.26**
Mouse 1R		**5**	95	600	∞	∞	95	2000	190001	5.28			
Mouse 2L		5	51	300	∞	∞	51	2000	102001	5.01			
Mouse 2R		3	46	220	∞	∞	46	2000	92001	4.96			
Mouse 3L		**10**	55	300	∞	∞	55	2000	110001	5.04			
Mouse 3R		20	190	∞	∞	∞	20	20000	400001	5.60			

Week 1 RIF$_{20}$-CFZ$_{12.5}$

Footpads	**all plain**						avg cfu ct	dilution facto	(X+1)	log cfu		**mean log cfu**	**st dev**
		**-4**	**-3**	**-2**	**-1**	**neat**							
Mouse 1L		9	70	400	∞	∞+	70	2000	140001	5.15		**5.39**	**0.36**
Mouse 1R		14	130	550	∞	∞+	14	20000	280001	5.45			
Mouse 2L		5	52	400	∞	∞+	52	2000	104001	5.02			
Mouse 2R		13	60	220	∞	∞+	13	20000	260001	5.41			
Mouse 3L									1				
Mouse 3R		44	300	∞	∞+	∞+	44	20000	880001	5.94			

Week 1 RIF$_{40}$-CFZ$_{12.5}$

Footpads	**all plain**						avg cfu ct	dilution facto	(X+1)	log cfu		**mean log cfu**	**st dev**
		**-4**	**-3**	**-2**	**-1**	**neat**							
Mouse 1L		2	26	400	∞	∞+	26	2000	52001	4.72		**4.66**	**0.32**
Mouse 1R		1	20	200	∞	∞+	20	2000	40001	4.60			
Mouse 2L		3	45	200	∞	∞+	45	2000	90001	4.95			
Mouse 2R		0	12	56	500	∞+	56	200	11201	4.05			
Mouse 3L		4	32	120	1000	∞+	32	2000	64001	4.81			
Mouse 3R		1	35	150	1000	∞+	35	2000	70001	4.85			

Week 1 RPT$_{10}$-CFZ$_{12.5}$

Footpads	**all plain**						avg cfu ct	dilution facto	(X+1)	log cfu		**mean log cfu**	**st dev**
		**-4**	**-3**	**-2**	**-1**	**neat**							
Mouse 1L		0	11	110	500	∞+	11	2000	22001	4.34		**4.57**	**0.20**
Mouse 1R		1	16	100	500	∞+	16	2000	32001	4.51			
Mouse 2L		3	36	100	800	∞+	36	2000	72001	4.86			

Mouse 2R		1	14	100	1000	∞+	14	2000	28001	4.45			
Mouse 3L		1	30	200	900	∞+	30	2000	60001	4.78			
Mouse 3R		1	15	150	800	∞+	15	2000	30001	4.48			

Week 1 RPT$_{20}$-CFZ$_{12.5}$

Footpads	**all plain**						avg cfu ct	dilution factor	(X+1)	log cfu		**mean log cfu**	**st dev**
		**-4**	**-3**	**-2**	**-1**	**neat**							
Mouse 1L		1	34	140	1200	∞	34	2000	68001	4.83		**4.20**	**0.86**
Mouse 1R		2	8	88	400	∞	88	200	17601	4.25			
Mouse 2L		2	33	150	1000	∞	33	2000	66001	4.82			
Mouse 2R		7	43	200	1200	∞	43	2000	86001	4.93			
Mouse 3L		0	0	9	31	23	31	20	621	2.79			
Mouse 3R		0	4	18	84	100	18	200	3601	3.56			

	A	B	C	D	E	F	G	H	I	J	K	L	M	N
1	**Week 2**	Untreated												
2	Footpads	**all plain**						avg cfu ct	dilution facto	(X+1)	log cfu		**mean log cfu**	**st dev**
3			**-4**	**-3**	**-2**	**-1**	**neat**							
4	Mouse 1L	3	70	600	∞		∞+	70	30000	2100001	6.32		**6.36**	**0.05**
5	Mouse 1R	9	68	550	∞		∞+	68	30000	2040001	6.31			
6	Mouse 2L	15	83	750	∞		∞+	83	30000	2490001	6.40			
7	Mouse 2R	8	92	850	∞		∞+	92	30000	2760001	6.44			
8	Mouse 3L	15	68	500	∞		∞+	68	30000	2040001	6.31			
9	Mouse 3R	18	80	800	∞		∞+	80	30000	2400001	6.38			
10														
11	**Week 2**	RIF-STR												
12	Footpads	**all plain**						avg cfu ct	dilution facto	(X+1)	log cfu		**mean log cfu**	**st dev**
13			**-4**	**-3**	**-2**	**-1**	**neat**							
14	Mouse 1L			0	6	50	500	50	30	1501	3.18		**3.99**	**0.43**
15	Mouse 1R			1	33	300	∞	33	300	9901	4.00			
16	Mouse 2L			6	57	500	∞	57	300	17101	4.23			
17	Mouse 2R			4	26	250	∞	26	300	7801	3.89			
18	Mouse 3L			10	78	800	∞	78	300	23401	4.37			
19	Mouse 3R			6	*53*	500	∞	6	3000	18001	4.26			
20					*agar shrank (E19)*									
21	**Week 2**	RIF-CLR												
22	Footpads	**all plain**						avg cfu ct	dilution facto	(X+1)	log cfu		**mean log cfu**	**st dev**
23			**-4**	**-3**	**-2**	**-1**	**neat**							
24	Mouse 1L			21	200	∞	∞+	21	3000	63001	4.80		**4.90**	**0.20**
25	Mouse 1R			26	240	∞	∞+	26	3000	78001	4.89			
26	Mouse 2L			43	400	∞	∞+	43	3000	129001	5.11			
27	Mouse 2R			14	150	∞	∞+	14	3000	42001	4.62			
28	Mouse 3L			23	250	∞	∞+	23	3000	69001	4.84			
29	Mouse 3R			46	400	∞	∞+	46	3000	138001	5.14			
30														
31	**Week 2**	RIF$_{10}$-CFZ$_{25}$												
32	Footpads	**all plain**						avg cfu ct	dilution facto	(X+1)	log cfu		**mean log cfu**	**st dev**
33			**-4**	**-3**	**-2**	**-1**	**neat**							
34	Mouse 1L			20	180	∞	∞+	20	3000	60001	4.78		**5.16**	**0.23**
35	Mouse 1R			59	450	∞	∞+	59	3000	177001	5.25			
36	Mouse 2L			37	400	∞	∞+	37	3000	111001	5.05			
37	Mouse 2R			72	600	∞	∞+	72	3000	216001	5.33			
38	Mouse 3L			44	400	∞	∞+	44	3000	132001	5.12			
39	Mouse 3R			85	750	∞	∞+	85	3000	255001	5.41			
40														
41	**Week 2**	RIF$_{10}$												
42	Footpads	**all plain**						avg cfu ct	dilution facto	(X+1)	log cfu		**mean log cfu**	**st dev**
43			**-4**	**-3**	**-2**	**-1**	**neat**							
44	Mouse 1L			111	900	∞	∞+	111	3000	333001	5.52		**5.58**	**0.23**
45	Mouse 1R			90	1000	∞	∞+	90	3000	270001	5.43			
46	Mouse 2L			112	1000	∞	∞+	112	3000	336001	5.53			

	A	B	C	D	E	F	G	H	I	J	K	L	M	N
47	Mouse 2R			83	800	∞	∞+	83	3000	249001	5.40			
48	Mouse 3L			124	1000	∞	∞+	124	3000	372001	5.57			
49	Mouse 3R			350	∞	∞+	∞++	350	3000	1050001	6.02			
50														
51	**Week 2**	RIF$_{20}$												
52	Footpads	**all plain**						avg cfu ct	dilution facto	(X+1)	log cfu		**mean log cfu**	**st dev**
53			**-4**	**-3**	**-2**	**-1**	**neat**							
54	Mouse 1L			32	150	1200	∞	32	3000	96001	4.98		**4.93**	**0.21**
55	Mouse 1R		plate->	5	115	400	∞	115	300	34501	4.54			
56	Mouse 2L			33	200	900	∞	33	3000	99001	5.00			
57	Mouse 2R			41	350	∞	∞+	41	3000	123001	5.09			
58	Mouse 3L			24	220	1000	∞	24	3000	72001	4.86			
59	Mouse 3R			44	400	5	∞+	44	3000	132001	5.12			
60														
61	**Week 2**	RIF$_{40}$												
62	Footpads	**all plain**						avg cfu ct	dilution facto	(X+1)	log cfu		**mean log cfu**	**st dev**
63			**-4**	**-3**	**-2**	**-1**	**neat**							
64	Mouse 1L			5	29	150	∞	29	300	8701	3.94		**4.38**	**0.52**
65	Mouse 1R			3	56	200	∞	56	300	16801	4.23			
66	Mouse 2L			22	73	750	∞	22	3000	66001	4.82			
67	Mouse 2R			7	71	240	∞	71	300	21301	4.33			
68	Mouse 3L			22	200	∞	∞+	22	300	6601	3.82			
69	Mouse 3R			49	350	2000	∞	49	3000	147001	5.17			
70														
71	**Week 2**	RPT$_{10}$												
72	Footpads	**all plain**						avg cfu ct	dilution facto	(X+1)	log cfu		**mean log cfu**	**st dev**
73			**-4**	**-3**	**-2**	**-1**	**neat**							
74	Mouse 1L			1	27	150	1000	27	300	8101	3.91		**3.53**	**0.40**
75	Mouse 1R			2	21	150	800	21	300	6301	3.80			
76	Mouse 2L			1	14	100	1000	14	300	4201	3.62			
77	Mouse 2R			2	9	100	750	100	30	3001	3.48			
78	Mouse 3L			0	5	20	150	20	30	601	2.78			
79	Mouse 3R			1	13	80	450	13	300	3901	3.59			
80														
81	**Week 2**	RPT$_{20}$												
82	Footpads	**all plain**						avg cfu ct	dilution facto	(X+1)	log cfu		**mean log cfu**	**st dev**
83			**-4**	**-3**	**-2**	**-1**	**neat**							
84	Mouse 1L			12	85	450	∞	12	3000	36001	4.56		**4.40**	**0.46**
85	Mouse 1R			20	100	550	∞	20	3000	60001	4.78			
86	Mouse 2L			20	59	700	∞	20	3000	60001	4.78			
87	Mouse 2R			15	31	400	∞	15	3000	45001	4.65			
88	Mouse 3L			3	24	90	1000	24	300	7201	3.86			
89	Mouse 3R			3	20	100	1000	20	300	6001	3.78			
90														
91	**Week 2**	RIF$_{10}$-CFZ$_{12.5}$												
92	Footpads	**all plain**						avg cfu ct	dilution facto	(X+1)	log cfu		**mean log cfu**	**st dev**

	A	B	C	D	E	F	G	H	I	J	K	L	M	N
93			**-4**	**-3**	**-2**	**-1**	**neat**							
94	Mouse 1L			80	600	∞	∞+	80	3000	240001	5.38		**5.14**	**0.28**
95	Mouse 1R			57	450	∞	∞+	57	3000	171001	5.23			
96	Mouse 2L			34	300	∞	∞+	34	3000	102001	5.01			
97	Mouse 2R			62	500	∞	∞+	62	3000	186001	5.27			
98	Mouse 3L			14	110	800	∞+	14	3000	42001	4.62			
99	Mouse 3R			66	500	∞	∞+	66	3000	198001	5.30			
100														
101	**Week 2**	RIF$_{20}$-CFZ$_{12.5}$												
102	Footpads	**all plain**						avg cfu ct	dilution facto	(X+1)	log cfu		**mean log cfu**	**st dev**
103			**-4**	**-3**	**-2**	**-1**	**neat**							
104	Mouse 1L			20	150∞	∞	∞+	20	3000	60001	4.78		**5.05**	**0.23**
105	Mouse 1R			60	600	∞	∞+	60	3000	180001	5.26			
106	Mouse 2L			61	600	∞	∞+	61	3000	183001	5.26			
107	Mouse 2R			47	400	∞	∞+	47	3000	141001	5.15			
108	Mouse 3L			18	200	∞	∞+	18	3000	54001	4.73			
109	Mouse 3R			42	350	∞	∞+	42	3000	126001	5.10			
110														
111	**Week 2**	RIF$_{40}$-CFZ$_{12.5}$												
112	Footpads	**all plain**						avg cfu ct	dilution facto	(X+1)	log cfu		**mean log cfu**	**st dev**
113			**-4**	**-3**	**-2**	**-1**	**neat**							
114	Mouse 1L			2	27	260	∞	27	300	8101	3.91		**4.05**	**0.66**
115	Mouse 1R			62	600	∞	∞+	62	3000	186001	5.27			
116	Mouse 2L			3	43	300	∞	43	300	12901	4.11			
117	Mouse 2R			0	6	100	∞	100	30	3001	3.48			
118	Mouse 3L			1	10	100	∞	100	30	3001	3.48			
119	Mouse 3R			4	38	170	∞	38	300	11401	4.06			
120														
121	**Week 2**	RPT$_{10}$-CFZ$_{12.5}$												
122	Footpads	**all plain**						avg cfu ct	dilution facto	(X+1)	log cfu		**mean log cfu**	**st dev**
123			**-4**	**-3**	**-2**	**-1**	**neat**							
124	Mouse 1L			1	34	90	∞	34	300	10201	4.01		**3.83**	**0.50**
125	Mouse 1R			5	10	90	∞	90	30	2701	3.43			
126	Mouse 2L			11	90	150	∞	90	300	27001	4.43			
127	Mouse 2R			1	0	37	300	37	30	1111	3.05			
128	Mouse 3L			3	30	80	∞	30	300	9001	3.95			
129	Mouse 3R			3	40	200	∞	40	300	12001	4.08			
130														
131	**Week 2**	RPT$_{20}$-CFZ$_{12.5}$												
132	Footpads	**all plain**						avg cfu ct	dilution facto	(X+1)	log cfu		**mean log cfu**	**st dev**
133			**-4**	**-3**	**-2**	**-1**	**neat**							
134	Mouse 1L			1	9	72	∞	72	30	2161	3.33		**3.36**	**0.24**
135	Mouse 1R			1	2	33	350	33	30	991	3.00			
136	Mouse 2L			2	6	65	500	65	30	1951	3.29			
137	Mouse 2R			1	7	65	450	65	30	1951	3.29			
138	Mouse 3L			4	15	100	∞	15	300	4501	3.65			

	A	B	C	D	E	F	G	H	I	J	K	L	M	N
139	Mouse 3R			0	13	93	1000	13	300	3901	3.59			

	A	B	C	D	E	F	G	H	I	J	K	L	M	N
1	**Week 4**	Untreated												
2	Footpads	**all plain**						avg cfu ct	dilution facto	(X+1)	log cfu		**mean log cfu**	**st dev**
3			**-4**	**-3**	**-2**	**-1**	**neat**							
4	Mouse 1L		x	x	x	x	x	x	30000	#VALUE!	#VALUE!		**#VALUE!**	**#VALUE!**
5	Mouse 1R		x	x	x	x	x	x	30000	#VALUE!	#VALUE!			
6	Mouse 2L		x	x	x	x	x	x	30000	#VALUE!	#VALUE!			
7	Mouse 2R		x	x	x	x	x	x	30000	#VALUE!	#VALUE!			
8	Mouse 3L		x	x	x	x	x	x	30000	#VALUE!	#VALUE!			
9	Mouse 3R		x	x	x	x	x	x	30000	#VALUE!	#VALUE!			
10														
11	**Week 4**	RIF-STR												
12	Footpads	**all plain**						avg cfu ct	dilution facto	(X+1)	log cfu		**mean log cfu**	**st dev**
13			**-4**	**-3**	**-2**	**-1**	**neat**							
14	Mouse 1L				0	0	5	5	3	16	1.20		**0.61**	**0.72**
15	Mouse 1R				0	0	14	14	3	43	1.63			
16	Mouse 2L				0	0	0	0	3	1	0.00		#positive	3
17	Mouse 2R				0	0	0	0	3	1	0.00		total	6
18	Mouse 3L				0	0	0	0	3	1	0.00			
19	Mouse 3R				0	0	2	2	3	7	0.85			
20														
21	**Week 4**	RIF-CLR												
22	Footpads	**all plain**						avg cfu ct	dilution facto	(X+1)	log cfu		**mean log cfu**	**st dev**
23			**-4**	**-3**	**-2**	**-1**	**neat**							
24	Mouse 1L				0	0	3	3	3	10	1.00		**0.97**	**0.26**
25	Mouse 1R				0	1	3	3	3	10	1.00			
26	Mouse 2L				0	0	3	3	3	10	1.00		#positive	6
27	Mouse 2R				0	0	8	8	3	25	1.40		total	6
28	Mouse 3L				0	0	1	1	3	4	0.60			
29	Mouse 3R				0	0	2	2	3	7	0.85			
30														
31	**Week 4**	RIF$_{10}$-CFZ$_{25}$												
32	Footpads	**all plain**						avg cfu ct	dilution facto	(X+1)	log cfu		**mean log cfu**	**st dev**
33			**-4**	**-3**	**-2**	**-1**	**neat**							
34	Mouse 1L				0	1	8	8	3	25	1.40		**2.41**	**0.92**
35	Mouse 1R				0	4	69	69	3	208	2.32			
36	Mouse 2L				28	230	1000	28	300	8401	3.92		#positive	6
37	Mouse 2R				0	4	35	35	3	106	2.03		total	6
38	Mouse 3L				0	1	21	21	3	64	1.81			
39	Mouse 3R				4	34	300	34	30	1021	3.01			
40														
41	**Week 4**	RIF$_{10}$												
42	Footpads	**all plain**						avg cfu ct	dilution facto	(X+1)	log cfu		**mean log cfu**	**st dev**
43			**-4**	**-3**	**-2**	**-1**	**neat**							
44	Mouse 1L			22	140	1000	∞	22	3000	66001	4.82		**4.40**	**0.95**
45	Mouse 1R			1	7	65	1000	65	30	1951	3.29			
46	Mouse 2L			98	800	∞	∞+	98	3000	294001	5.47		#positive	4

	A	B	C	D	E	F	G	H	I	J	K	L	M	N
47	Mouse 2R			8	36	250	∞	36	300	10801	4.03		total	6
48	Mouse 3L						x	0	3000	1				
49	Mouse 3R						x	0	3000	1				
50														
51	**Week 4**	RIF$_{20}$												
52	Footpads	**all plain**						avg cfu ct	dilution facto	(X+1)	log cfu		**mean log cfu**	**st dev**
53			**-4**	**-3**	**-2**	**-1**	**neat**							
54	Mouse 1L				0	1	6	6	3	19	1.28		**0.87**	**0.56**
55	Mouse 1R				0	0	8	8	3	25	1.40			
56	Mouse 2L				0	0	7	7	3	22	1.34		#positive	5
57	Mouse 2R				0	1	1	1	3	4	0.60		total	6
58	Mouse 3L				0	0	0	0	3	1	0.00			
59	Mouse 3R				0	1	1	1	3	4	0.60			
60														
61	**Week 4**	RIF$_{40}$												
62	Footpads	**all plain**						avg cfu ct	dilution facto	(X+1)	log cfu		**mean log cfu**	**st dev**
63			**-4**	**-3**	**-2**	**-1**	**neat**							
64	Mouse 1L				0	0	2	2	3	7	0.85		**0.69**	**0.68**
65	Mouse 1R				0	0	22	22	3	67	1.83			
66	Mouse 2L				0	0	0	0	3	1	0.00		#positive	4
67	Mouse 2R				0	0	2	2	3	7	0.85		total	6
68	Mouse 3L				0	0	0	0	3	1	0.00			
69	Mouse 3R				0	0	1	1	3	4	0.60			
70														
71	**Week 4**	RPT$_{10}$												
72	Footpads	**all plain**						avg cfu ct	dilution facto	(X+1)	log cfu		**mean log cfu**	**st dev**
73			**-4**	**-3**	**-2**	**-1**	**neat**							
74	Mouse 1L				0	0	0	0	3	1	0.00		**0.17**	**0.41**
75	Mouse 1R				0	0	0	0	3	1	0.00			
76	Mouse 2L				0	0	0	0	3	1	0.00		#positive	1
77	Mouse 2R				0	1	3	3	3	10	1.00		total	6
78	Mouse 3L				0	0	0	0	3	1	0.00			
79	Mouse 3R				0	0	0	0	3	1	0.00			
80														
81	**Week 4**	RPT$_{20}$												
82	Footpads	**all plain**						avg cfu ct	dilution facto	(X+1)	log cfu		**mean log cfu**	**st dev**
83			**-4**	**-3**	**-2**	**-1**	**neat**							
84	Mouse 1L				0	0	0	0	3	1	0.00		**0.00**	**0.00**
85	Mouse 1R				0	0	0	0	3	1	0.00			
86	Mouse 2L				0	0	0	0	3	1	0.00		#positive	0
87	Mouse 2R				0	0	0	0	3	1	0.00		total	6
88	Mouse 3L				0	0	0	0	3	1	0.00			
89	Mouse 3R				0	0	0	0	3	1	0.00			
90														
91	**Week 4**	RIF$_{10}$-CFZ$_{12.5}$												
92	Footpads	**all plain**						avg cfu ct	dilution facto	(X+1)	log cfu		**mean log cfu**	**st dev**

	A	B	C	D	E	F	G	H	I	J	K	L	M	N
93			**-4**	**-3**	**-2**	**-1**	**neat**							
94	Mouse 1L				0	0	0	0	3	1	0.00		**0.14**	**0.35**
95	Mouse 1R				0	0	0	0	3	1	0.00			
96	Mouse 2L				0	0	0	0	3	1	0.00		#positive	1
97	Mouse 2R				0	0	2	2	3	7	0.85		total	6
98	Mouse 3L				0	0	0	0	3	1	0.00			
99	Mouse 3R				0	0	0	0	3	1	0.00			
100														
101	**Week 4**	RIF$_{20}$-CFZ$_{12.5}$												
102	Footpads	**all plain**						avg cfu ct	dilution facto	(X+1)	log cfu		**mean log cfu**	**st dev**
103			**-4**	**-3**	**-2**	**-1**	**neat**							
104	Mouse 1L				0	0	0	0	3	1	0.00		**0.42**	**0.67**
105	Mouse 1R				0	0	0	0	3	1	0.00			
106	Mouse 2L				0	2	11	11	3	34	1.53		#positive	2
107	Mouse 2R				0	0	3	3	3	10	1.00	**used charco	total	6
108	Mouse 3L				0	0	0	0	3	1	0.00			
109	Mouse 3R				0	0	0	0	3	1	0.00			
110														
111	**Week 4**	RIF$_{40}$-CFZ$_{12.5}$												
112	Footpads	**all plain**						avg cfu ct	dilution facto	(X+1)	log cfu		**mean log cfu**	**st dev**
113			**-4**	**-3**	**-2**	**-1**	**neat**							
114	Mouse 1L				0	0	0	0	3	1	0.00		**0.00**	**0.00**
115	Mouse 1R				0	0	0	0	3	1	0.00			
116	Mouse 2L				0	0	0	0	3	1	0.00		#positive	0
117	Mouse 2R				0	0	0	0	3	1	0.00		total	6
118	Mouse 3L				0	0	0	0	3	1	0.00			
119	Mouse 3R				0	0	0	0	3	1	0.00			
120														
121	**Week 4**	RPT$_{10}$-CFZ$_{12.5}$												
122	Footpads	**all plain**						avg cfu ct	dilution facto	(X+1)	log cfu		**mean log cfu**	**st dev**
123			**-4**	**-3**	**-2**	**-1**	**neat**							
124	Mouse 1L				0	0	0	0	3	1	0.00		**0.00**	**0.00**
125	Mouse 1R				0	0	0	0	3	1	0.00			
126	Mouse 2L				0	0	0	0	3	1	0.00		#positive	0
127	Mouse 2R				0	0	0	0	3	1	0.00		total	6
128	Mouse 3L				0	0	0	0	3	1	0.00			
129	Mouse 3R				0	0	0	0	3	1	0.00			
130														
131	**Week 4**	RPT$_{20}$-CFZ$_{12.5}$												
132	Footpads	**all plain**						avg cfu ct	dilution facto	(X+1)	log cfu		**mean log cfu**	**st dev**
133			**-4**	**-3**	**-2**	**-1**	**neat**							
134	Mouse 1L				0	0	0	0	3	1	0.00		**0.10**	**0.25**
135	Mouse 1R				0	0	0	0	3	1	0.00			
136	Mouse 2L				0	0	0	0	3	1	0.00		#positive	1
137	Mouse 2R				0	0	0	0	3	1	0.00		total	6
138	Mouse 3L				0	0	1	1	3	4	0.60			

	A	B	C	D	E	F	G	H	I	J	K	L	M	N
139	Mouse 3R				0	0	0	0	3	1	0.00			

	A	B	C	D	E	F	G	H	I	J	K	L
2	**Wk 4+ 3M**	RIF-STR										
3	Footpads	**all plain**					avg cfu ct	dilution facto	(X+1)	log cfu	**mean log cfu**	**st dev**
4			**-2**	**-1**	**UD1**	**UD2**						
5	Mouse 1L		0	0	0	0	0	3	1	0.00	**2.58**	**2.43**
6	Mouse 1R		2000				2000	300	600001	5.78		
7	Mouse 2L		2000				2000	300	600001	5.78	min	0.00
8	Mouse 2R		100				100	300	30001	4.48	max	5.78
9	Mouse 3L		120				120	300	36001	4.56		
10	Mouse 3R		45				45	300	13501	4.13	median	2.54
11	Mouse 4L		0	0	0	0	0	3	1	0.00		
12	Mouse 4R		0	0	0	0	0	3	1	0.00	#positive	12
13	Mouse 5L		0	0	0	0	0	3	1	0.00	total	20
14	Mouse 5R		0	0	0	0	0	3	1	0.00		
15	Mouse 6L		2000				2000	300	600001	5.78		
16	Mouse 6R		7	32	65	200 c	32	30	961	2.98		
17	Mouse 7L		55	500			55	300	16501	4.22		
18	Mouse 7R		0	1	6	12	9	3	28	1.45		
19	Mouse 8L		300				300	300	90001	4.95		
20	Mouse 8R		1000				1000	300	300001	5.48		
21	Mouse 9L		1	5	40	41	41	3	123	2.09		
22	Mouse 9R		0	0	0	0	0	3	1	0.00		
23	Mouse 10L		0	0	0	0	0	3	1	0.00		
24	Mouse 10R		0	0	0	0	0	3	1	0.00		
25												
26												
27	**Wk 4+ 3M**	RIF-CLR										
28	Footpads	**all plain**					avg cfu ct	dilution facto	(X+1)	log cfu	**mean log cfu**	**st dev**
29			**-2**	**-1**	**UD1**	**UD2**						
30	Mouse 1L		100				100	300	30001	4.48	**3.34**	**2.11**
31	Mouse 1R		200				200	300	60001	4.78		
32	Mouse 2L		1000				1000	300	300001	5.48	min	0.00
33	Mouse 2R		1000				1000	300	300001	5.48	max	5.78
34	Mouse 3L		0		0	0	0	3	1	0.00		
35	Mouse 3R		0	2	3	1	2	3	7	0.85	median	4.13
36	Mouse 4L		6	33	200	200	33	3	100	2.00		
37	Mouse 4R		1	2	26	24	25	3	76	1.88	#positive	16

	A	B	C	D	E	F	G	H	I	J	K	L
38	Mouse 5L										total	18
39	Mouse 5R											
40	Mouse 6L		2000				2000	300	600001	5.78		
41	Mouse 6R		500				500	300	150001	5.18		
42	Mouse 7L		0	0	1	1	1	3	4	0.60		
43	Mouse 7R		0	0	0	0	0	3	1	0.00		
44	Mouse 8L		19	200	∞	∞	200	30	6001	3.78		
45	Mouse 8R		0	1	7	5	6	3	19	1.28		
46	Mouse 9L		100				100	300	30001	4.48		
47	Mouse 9R		200				200	300	60001	4.78		
48	Mouse 10L		14	100			100	30	3001	3.48		
49	Mouse 10R		2000				2000	300	600001	5.78		
50												
51												
52	**Wk 4+ 3M**	RIF$_{10}$-CFZ$_{25}$										
53	Footpads	**all plain**					avg cfu ct	dilution facto	(X+1)	log cfu	**mean log cfu**	**st dev**
54			**-2**	**-1**	**UD1**	**UD2**						
55	Mouse 1L		1000	∞	lawn	lawn	1000	300	300001	5.48	**1.31**	**1.99**
56	Mouse 1R		300	3000	∞	∞	300	300	90001	4.95		
57	Mouse 2L		0	1	30	36	33	3	100	2.00	min	0.00
58	Mouse 2R		300	∞	∞+	∞+	300	300	90001	4.95	max	5.48
59	Mouse 3L		3	23	250	250	23	30	691	2.84		
60	Mouse 3R		0	22	100	300	22	30	661	2.82	median	0.00
61	Mouse 4L		0	0	0	0	0	3	1	0.00		
62	Mouse 4R		0	0	0	0	0	3	1	0.00	#positive	7
63	Mouse 5L		0	0	0	0	0	3	1	0.00	total	20
64	Mouse 5R		4	54	250	250	54	30	1621	3.21		
65	Mouse 6L		0	0	0	0	0	3	1	0.00		
66	Mouse 6R		0	0	0	0	0	3	1	0.00		
67	Mouse 7L		0	0	0	0	0	3	1	0.00		
68	Mouse 7R		0	0	0	0	0	3	1	0.00		
69	Mouse 8L		0	0	0	0	0	3	1	0.00		
70	Mouse 8R		0	0	0	0	0	3	1	0.00		
71	Mouse 9L		0	0	0	0	0	3	1	0.00		
72	Mouse 9R		0	0	0	0	0	3	1	0.00		
73	Mouse 10L		0	0	0	0	0	3	1	0.00		

	A	B	C	D	E	F	G	H	I	J	K	L
74	Mouse 10R		0	0	0	0	0	3	1	0.00		
75												
76												
77	**Wk 4+ 3M**	RIF$_{10}$-CFZ$_{12.5}$										
78	Footpads	**all plain**					avg cfu ct	dilution facto	(X+1)	log cfu	**mean log cfu**	**st dev**
79			**-2**	**-1**	**UD1**	**UD2**						
80	Mouse 1L		0	0	0	0	0	3	1	0.00	**1.17**	**1.81**
81	Mouse 1R		0	0	0	0	0	3	1	0.00		
82	Mouse 2L		0	0	4	4	4	3	13	1.11	min	0.00
83	Mouse 2R		0	0	0	0	0	3	1	0.00	max	5.78
84	Mouse 3L		0	0	0	0	0	3	1	0.00		
85	Mouse 3R		0	0	12	1	7	3	21	1.31	median	0.00
86	Mouse 4L		0	0	2	7	5	3	15	1.16		
87	Mouse 4R		0	2	11	7	9	3	28	1.45	#positive	9
88	Mouse 5L		2000	∞+	lawn	lawn	2000	300	600001	5.78	total	20
89	Mouse 5R		0	8	19	29	24	3	73	1.86		
90	Mouse 6L		0	0	0	0	0	3	1	0.00		
91	Mouse 6R		0	0	0	0	0	3	1	0.00		
92	Mouse 7L		0	0	0	0	0	3	1	0.00		
93	Mouse 7R		0	0	0	0	0	3	1	0.00		
94	Mouse 8L		0	0	0	0	0	3	1	0.00		
95	Mouse 8R		4	9	300	300	300	3	901	2.95		
96	Mouse 9L		0	0	0	0	0	3	1	0.00		
97	Mouse 9R		0	0	0	0	0	3	1	0.00		
98	Mouse 10L		2000	∞+	lawn	lawn	2000	300	600001	5.78		
99	Mouse 10R		0	2	35	41	38	3	115	2.06		
100												
101	**Wk 4+ 3M**	RIF$_{20}$-CFZ$_{12.5}$										
102	Footpads	**all plain**					avg cfu ct	dilution facto	(X+1)	log cfu	**mean log cfu**	**st dev**
103			**-2**	**-1**	**UD1**	**UD2**						
104	Mouse 1L		0	0	0	0	0	3	1	0.00	**0.00**	**0.00**
105	Mouse 1R		0	0	0	0	0	3	1	0.00		
106	Mouse 2L		0	0	0	0	0	3	1	0.00		
107	Mouse 2R		0	0	0	0	0	3	1	0.00	min	0.00
108	Mouse 3L		0	0	0	0	0	3	1	0.00	max	0.00
109	Mouse 3R		0	0	0	0	0	3	1	0.00		

	A	B	C	D	E	F	G	H	I	J	K	L
110	Mouse 4L		0	0	0	0	0	3	1	0.00	median	0.00
111	Mouse 4R		0	0	0	0	0	3	1	0.00		
112	Mouse 5L		0	0	0	0	0	3	1	0.00	#positive	0
113	Mouse 5R		0	0	0	0	0	3	1	0.00	total	16
114	Mouse 6L		0	0	0	0	0	3	1	0.00		
115	Mouse 6R		0	0	0	0	0	3	1	0.00		
116	Mouse 7L		0	0	0	0	0	3	1	0.00		
117	Mouse 7R		0	0	0	0	0	3	1	0.00		
118	Mouse 8L		0	0	0	0	0	3	1	0.00		
119	Mouse 8R		0	0	0	0	0	3	1	0.00		
120	Mouse 9L											
121	Mouse 9R											
122	Mouse 10L											
123	Mouse 10R											
124												
125	**Wk 4+ 3M**	RIF$_{40}$-CFZ$_{12.5}$										
126	Footpads	**all plain**					avg cfu ct	dilution facto	(X+1)	log cfu	**mean log cfu**	**st dev**
127			**-2**	**-1**	**UD1**	**UD2**						
128	Mouse 1L			0	0	0	0	3	1	0.00	**0.00**	**0.00**
129	Mouse 1R			0	0	0	0	3	1	0.00		
130	Mouse 2L			0	0	0	0	3	1	0.00		
131	Mouse 2R			0	0	0	0	3	1	0.00	min	0.00
132	Mouse 3L			0	0	0	0	3	1	0.00	max	0.00
133	Mouse 3R			0	0	0	0	3	1	0.00		
134	Mouse 4L			0	0	0	0	3	1	0.00	median	0.00
135	Mouse 4R			0	0	0	0	3	1	0.00		
136	Mouse 5L			0	0	0	0	3	1	0.00	#positive	0
137	Mouse 5R			0	0	0	0	3	1	0.00	total	16
138	Mouse 6L			0	0	0	0	3	1	0.00		
139	Mouse 6R			0	0	0	0	3	1	0.00		
140	Mouse 7L			0	0	0	0	3	1	0.00		
141	Mouse 7R			0	0	0	0	3	1	0.00		
142	Mouse 8L			0	0	0	0	3	1	0.00		
143	Mouse 8R			0	0	0	0	3	1	0.00		
144	Mouse 9L											
145	Mouse 9R											

	A	B	C	D	E	F	G	H	I	J	K	L
146	Mouse 10L											
147	Mouse 10R											
148												
149	**Wk 4+ 3M**	RPT$_{10}$-CFZ$_{12.5}$										
150	Footpads	**all plain**					avg cfu ct	dilution factoı	(X+1)	log cfu	**mean log cfu**	**st dev**
151			**-2**	**-1**	**UD1**	**UD2**						
152	Mouse 1L			0	0	0	0	3	1	0.00	**0.29**	**0.83**
153	Mouse 1R			0	0	0	0	3	1	0.00		
154	Mouse 2L			0	0	0	0	3	1	0.00		
155	Mouse 2R			0	0	0	0	3	1	0.00	min	0.00
156	Mouse 3L			0	0	0	0	3	1	0.00	max	3.48
157	Mouse 3R			0	0	0	0	3	1	0.00		
158	Mouse 4L			0	0	0	0	3	1	0.00	median	0.00
159	Mouse 4R			0	0	0	0	3	1	0.00		
160	Mouse 5L			0	0	0	0	3	1	0.00	#positive	3
161	Mouse 5R			0	0	0	0	3	1	0.00	total	20
162	Mouse 6L			100	1000	1000	1000	3	3001	3.48		
163	Mouse 6R			0	5	7	6	3	19	1.28		
164	Mouse 7L			0	2	4	3	3	10	1.00		
165	Mouse 7R			0	0	0	0	3	1	0.00		
166	Mouse 8L			0	0	0	0	3	1	0.00		
167	Mouse 8R			0	0	0	0	3	1	0.00		
168	Mouse 9L			0	0	0	0	3	1	0.00		
169	Mouse 9R			0	0	0	0	3	1	0.00		
170	Mouse 10L			0	0	0	0	3	1	0.00		
171	Mouse 10R			0	0	0	0	3	1	0.00		
172												
173	**Wk 4+ 3M**	RPT$_{20}$-CFZ$_{12.5}$										
174	Footpads	**all plain**					avg cfu ct	dilution factoı	(X+1)	log cfu	**mean log cfu**	**st dev**
175			**-2**	**-1**	**UD1**	**UD2**						
176	Mouse 1L			0	0	0	0	3	1	0.00	**0.00**	**0.00**
177	Mouse 1R			0	0	0	0	3	1	0.00		
178	Mouse 2L			0	0	0	0	3	1	0.00		
179	Mouse 2R			0	0	0	0	3	1	0.00	min	0.00
180	Mouse 3L			0	0	0	0	3	1	0.00	max	0.00
181	Mouse 3R			0	0	0	0	3	1	0.00		

	A	B	C	D	E	F	G	H	I	J	K	L
182	Mouse 4L			0	0	0	0	3	1	0.00	median	0.00
183	Mouse 4R			0	0	0	0	3	1	0.00		
184	Mouse 5L			0	0	0	0	3	1	0.00	#positive	0
185	Mouse 5R			0	0	0	0	3	1	0.00	total	20
186	Mouse 6L			0	0	0	0	3	1	0.00		
187	Mouse 6R			0	0	0	0	3	1	0.00		
188	Mouse 7L			0	0	0	0	3	1	0.00		
189	Mouse 7R			0	0	0	0	3	1	0.00		
190	Mouse 8L			0	0	0	0	3	1	0.00		
191	Mouse 8R			0	0	0	0	3	1	0.00		
192	Mouse 9L			0	0	0	0	3	1	0.00		
193	Mouse 9R			0	0	0	0	3	1	0.00		
194	Mouse 10L			0	0	0	0	3	1	0.00		
195	Mouse 10R			0	0	0	0	3	1	0.00		

	A	B	C	D	E	F	G	H	I	J	K	L
2	**Wk 6+ 3M**	RIF-STR										
3	Footpads	**all plain**					avg cfu ct	dilution factoı	(X+1)	log cfu	**mean log cfu**	**st dev**
4			**-2**	**-1**	**UD1**	**UD2**						
5	Mouse 1L			0	0	0	0	3	1	0.00	**0.51**	**1.43**
6	Mouse 1R			0	0	0	0	3	1	0.00		
7	Mouse 2L			0	0	0	0	3	1	0.00	min	0.00
8	Mouse 2R			0	0	0	0	3	1	0.00	max	4.78
9	Mouse 3L			0	0	0	0	3	1	0.00		
10	Mouse 3R			0	0	0	0	3	1	0.00	median	0.00
11	Mouse 4L			0	0	0	0	3	1	0.00		
12	Mouse 4R			0	0	0	0	3	1	0.00	#positive	3
13	Mouse 5L			0	0	0	0	3	1	0.00	total	20
14	Mouse 5R			0	0	0	0	3	1	0.00		
15	Mouse 6L			0	0	0	0	3	1	0.00		
16	Mouse 6R			0	0	0	0	3	1	0.00		
17	Mouse 7L			0	0	0	0	3	1	0.00		
18	Mouse 7R			2000	∞+	∞+	2000	30	60001	4.78		
19	Mouse 8L			0	0	0	0	3	1	0.00		
20	Mouse 8R			1000	∞+	∞+	1000	30	30001	4.48		
21	Mouse 9L			0	0	0	0	3	1	0.00		
22	Mouse 9R			1	2	4	3	3	10	1.00		
23	Mouse 10L			0	0	0	0	3	1	0.00		
24	Mouse 10R			0	0	0	0	3	1	0.00		
25												
26												
27	**Wk 6+ 3M**	RIF-CLR										
28	Footpads	**all plain**					avg cfu ct	dilution factoı	(X+1)	log cfu	**mean log cfu**	**st dev**
29			**-2**	**-1**	**UD1**	**UD2**						
30	Mouse 1L			0	0	0	0	3	1	0.00	**2.57**	**2.22**
31	Mouse 1R			0	0	0	0	3	1	0.00		
32	Mouse 2L			0	0	?	0	3	1	0.00	min	0.00
33	Mouse 2R			3	25	21	23	3	70	1.85	max	4.78
34	Mouse 3L			0	0	0	0	3	1	0.00		
35	Mouse 3R			0	0	0	0	3	1	0.00	median	2.67
36	Mouse 4L			2000	∞+	∞+	2000	30	60001	4.78		
37	Mouse 4R			2000	∞+	∞+	2000	30	60001	4.78	#positive	13
38	Mouse 5L			2000	∞+	∞+	2000	30	60001	4.78	total	20
39	Mouse 5R			2000	∞+	∞+	2000	30	60001	4.78		
40	Mouse 6L			0	1	??	0	30	1	0.00		
41	Mouse 6R			58	550	600	58	30	1741	3.24		
42	Mouse 7L			2000	∞+	∞+	2000	30	60001	4.78		
43	Mouse 7R			4	41	42	42	3	126	2.10		
44	Mouse 8L			0	7	7	7	2	15	1.18		
45	Mouse 8R			2000	∞+	∞+	2000	30	60001	4.78		
46	Mouse 9L			0	0	0	0	3	1	0.00		
47	Mouse 9R			2000	∞	+	2000	30	60001	4.78		
48	Mouse 10L			2000	∞	+	2000	30	60001	4.78		
49	Mouse 10R			2000	∞	+	2000	30	60001	4.78		
50												
51												
52	**Wk 6+ 3M**	RIF_{10}-CFZ_{25}										
53	Footpads	**all plain**					avg cfu ct	dilution factoı	(X+1)	log cfu	**mean log cfu**	**st dev**
54			**-2**	**-1**	**UD1**	**UD2**						
55	Mouse 1L			0	0	0	0	3	1	0.00	**0.00**	**0.00**
56	Mouse 1R			0	0	0	0	3	1	0.00		
57	Mouse 2L			0	0	0	0	3	1	0.00	min	0.00
58	Mouse 2R			0	0	0	0	3	1	0.00	max	0.00
59	Mouse 3L			0	0	0	0	3	1	0.00		
60	Mouse 3R			0	0	0	0	3	1	0.00	median	0.00
61	Mouse 4L			0	0	0	0	3	1	0.00		
62	Mouse 4R			0	0	0	0	3	1	0.00	#positive	0
63	Mouse 5L			0	0	0	0	3	1	0.00	total	16
64	Mouse 5R			0	0	0	0	3	1	0.00		
65	Mouse 6L			0	0	0	0	3	1	0.00		
66	Mouse 6R			0	0	0	0	3	1	0.00		
67	Mouse 7L			0	0	0	0	3	1	0.00		
68	Mouse 7R			0	0	0	0	3	1	0.00		
69	Mouse 8L			0	0	0	0	3	1	0.00		
70	Mouse 8R			0	0	0	0	3	1	0.00		

	A	B	C	D	E	F	G	H	I	J	K	L
71	Mouse 9L						0	3	1	0.00		
72	Mouse 9R						0	3	1	0.00		
73	Mouse 10L						0	3	1	0.00		
74	Mouse 10R						#DIV/0!	3	#DIV/0!	#DIV/0!		
75												
76												
77	**Wk 6+ 3M**	RIF$_{10}$-CFZ$_{12.5}$										
78	Footpads	**all plain**					avg cfu ct	dilution factoı	(X+1)	log cfu	**mean log cfu**	**st dev**
79			**-2**	**-1**	**UD1**	**UD2**						
80	Mouse 1L			0	0	0	0	3	1	0.00	**1.28**	**1.21**
81	Mouse 1R			0	0	0	0	3	1	0.00		
82	Mouse 2L			2	1	2	2	3	6	0.74	min	0.00
83	Mouse 2R			0	8	7	8	3	24	1.37	max	3.28
84	Mouse 3L			33	150	125	33	30	991	3.00		
85	Mouse 3R			4	33	16	25	3	75	1.87	median	1.43
86	Mouse 4L			1	0	0	1	30	31	1.49		
87	Mouse 4R			0	12	13	13	3	39	1.59	#positive	11
88	Mouse 5L			22	200	200	22	30	661	2.82	total	18
89	Mouse 5R			0	0	0	0	3	1	0.00		
90	Mouse 6L			64	250	225	64	30	1921	3.28		
91	Mouse 6R			16	125	150	16	30	481	2.68		
92	Mouse 7L			11	39	58	49	3	147	2.17		
93	Mouse 7R			6	30	34	32	3	97	1.99		
94	Mouse 8L			0	0	0	0	3	1	0.00		
95	Mouse 8R			0	0	0	0	3	1	0.00		
96	Mouse 9L			0	0	0	0	3	1	0.00		
97	Mouse 9R			0	0	0	0	3	1	0.00		
98	Mouse 10L						0	3	1	0.00		
99	Mouse 10R						#DIV/0!	3	#DIV/0!	#DIV/0!		
100												
101	**Wk 6+ 3M**	RIF$_{20}$-CFZ$_{12.5}$										
102	Footpads	**all plain**					avg cfu ct	dilution factoı	(X+1)	log cfu	**mean log cfu**	**st dev**
103			**-2**	**-1**	**UD1**	**UD2**						
104	Mouse 1L			0	0	0	0	3	1	0.00	**0.00**	**0.00**
105	Mouse 1R			0	0	0	0	3	1	0.00		
106	Mouse 2L			0	0	0	0	3	1	0.00		
107	Mouse 2R			0	0	0	0	3	1	0.00	min	0.00
108	Mouse 3L			0	0	0	0	3	1	0.00	max	0.00
109	Mouse 3R			0	0	0	0	3	1	0.00		
110	Mouse 4L			0	0	0	0	3	1	0.00	median	0.00
111	Mouse 4R			0	0	0	0	3	1	0.00		
112	Mouse 5L			0	0	0	0	3	1	0.00	#positive	0
113	Mouse 5R			0	0	0	0	3	1	0.00	total	20
114	Mouse 6L			0	0	0	0	3	1	0.00		
115	Mouse 6R			0	0	0	0	3	1	0.00		
116	Mouse 7L			0	0	0	0	3	1	0.00		
117	Mouse 7R			0	0	0	0	3	1	0.00		
118	Mouse 8L			0	0	0	0	3	1	0.00		
119	Mouse 8R			0	0	0	0	3	1	0.00		
120	Mouse 9L			0	0	0	0	3	1	0.00		
121	Mouse 9R			0	0	0	0	3	1	0.00		
122	Mouse 10L			0	0	0	0	3	1	0.00		
123	Mouse 10R			0	0	0	0	3	1	0.00		
124												
125	**Wk 6+ 3M**	RIF$_{40}$-CFZ$_{12.5}$										
126	Footpads	**all plain**					avg cfu ct	dilution factoı	(X+1)	log cfu	**mean log cfu**	**st dev**
127			**-2**	**-1**	**UD1**	**UD2**						
128	Mouse 1L			0	0	0	0	3	1	0.00	**0.00**	**0.00**
129	Mouse 1R			0	0	0	0	3	1	0.00		
130	Mouse 2L			0	0	0	0	3	1	0.00		
131	Mouse 2R			0	0	0	0	3	1	0.00	min	0.00
132	Mouse 3L			0	0	0	0	3	1	0.00	max	0.00
133	Mouse 3R			0	0	0	0	3	1	0.00		
134	Mouse 4L			0	0	0	0	3	1	0.00	median	0.00
135	Mouse 4R			0	0	0	0	3	1	0.00		
136	Mouse 5L			0	0	0	0	3	1	0.00	#positive	0
137	Mouse 5R			0	0	0	0	3	1	0.00	total	18
138	Mouse 6L			0	0	0	0	3	1	0.00		
139	Mouse 6R			0	0	0	0	3	1	0.00		

	A	B	C	D	E	F	G	H	I	J	K	L
140	Mouse 7L			0	0	0	0	3	1	0.00		
141	Mouse 7R			0	0	0	0	3	1	0.00		
142	Mouse 8L			0	0	0	0	3	1	0.00		
143	Mouse 8R			0	0	0	0	3	1	0.00		
144	Mouse 9L			0	0	0	0	3	1	0.00		
145	Mouse 9R			0	0	0	0	3	1	0.00		
146	Mouse 10L						0	3	1	0.00		
147	Mouse 10R						0	3	1	0.00		
148												
149	**Wk 6+ 3M**	RPT$_{10}$-CFZ$_{12.5}$										
150	Footpads	**all plain**					avg cfu ct	dilution factoı	(X+1)	log cfu	**mean log cfu**	**st dev**
151			**-2**	**-1**	**UD1**	**UD2**						
152	Mouse 1L			0	0	0	0	3	1	0.00	**0.00**	**0.00**
153	Mouse 1R			0	0	0	0	3	1	0.00		
154	Mouse 2L			0	0	0	0	3	1	0.00		
155	Mouse 2R			0	0	0	0	3	1	0.00	min	0.00
156	Mouse 3L			0	0	0	0	3	1	0.00	max	0.00
157	Mouse 3R			0	0	0	0	3	1	0.00		
158	Mouse 4L			0	0	0	0	3	1	0.00	median	0.00
159	Mouse 4R			0	0	0	0	3	1	0.00		
160	Mouse 5L			0	0	0	0	3	1	0.00	#positive	0
161	Mouse 5R			0	0	0	0	3	1	0.00	total	20
162	Mouse 6L			0	0	0	0	3	1	0.00		
163	Mouse 6R			0	0	0	0	3	1	0.00		
164	Mouse 7L			0	0	0	0	3	1	0.00		
165	Mouse 7R			0	0	0	0	3	1	0.00		
166	Mouse 8L			0	0	0	0	3	1	0.00		
167	Mouse 8R			0	0	0	0	3	1	0.00		
168	Mouse 9L			0	0	0	0	3	1	0.00		
169	Mouse 9R			0	0	0	0	3	1	0.00		
170	Mouse 10L			0	0	0	0	3	1	0.00		
171	Mouse 10R			0	0	0	0	3	1	0.00		
172												
173	**Wk 6+ 3M**	RPT$_{20}$-CFZ$_{12.5}$										
174	Footpads	**all plain**					avg cfu ct	dilution factoı	(X+1)	log cfu	**mean log cfu**	**st dev**
175			**-2**	**-1**	**UD1**	**UD2**						
176	Mouse 1L			0	0	0	0	3	1	0.00	**0.00**	**0.00**
177	Mouse 1R			0	0	0	0	3	1	0.00		
178	Mouse 2L			0	0	0	0	3	1	0.00		
179	Mouse 2R			0	0	0	0	3	1	0.00	min	0.00
180	Mouse 3L			0	0	0	0	3	1	0.00	max	0.00
181	Mouse 3R			0	0	0	0	3	1	0.00		
182	Mouse 4L			0	0	0	0	3	1	0.00	median	0.00
183	Mouse 4R			0	0	0	0	3	1	0.00		
184	Mouse 5L			0	0	0	0	3	1	0.00	#positive	0
185	Mouse 5R			0	0	0	0	3	1	0.00	total	20
186	Mouse 6L			0	0	0	0	3	1	0.00		
187	Mouse 6R			0	0	0	0	3	1	0.00		
188	Mouse 7L			0	0	0	0	3	1	0.00		
189	Mouse 7R			0	0	0	0	3	1	0.00		
190	Mouse 8L			0	0	0	0	3	1	0.00		
191	Mouse 8R			0	0	0	0	3	1	0.00		
192	Mouse 9L			0	0	0	0	3	1	0.00		
193	Mouse 9R			0	0	0	0	3	1	0.00		
194	Mouse 10L			0	0	0	0	3	1	0.00		
195	Mouse 10R			0	0	0	0	3	1	0.00		
