# Supplementary material for: Shorter-course treatment for Mycobacterium ulcerans disease with high-dose rifamycins and clofazimine in a mouse model of Buruli ulcer
Source: PLoS Negl Trop Dis. 2018 Aug 13;12(8):e0006728. doi: 10.1371/journal.pntd.0006728 (PMC6107292; doi:10.1371/journal.pntd.0006728)
Supplement: S5 Table — (PDF) [file pntd.0006728.s006.pdf]

Week 1	1L	2L	3L	1R	2R	3R			
	HMG	HMG	HMG	HMG	HMG	HMG			Mean log10
UT	101.8	108.6	86.47	108.7	108.2	103.7	102.91	8.56	2.01
RS	2.081	4.405	5.957	2.925	5.295	7.353	4.67	1.95	0.67
RCLR	28.56	5.499	11.83	15.76	7.983	6.917	12.76	8.59	1.11
R10C25		29.74	51.58		47.87	81.12	52.58	21.29	1.72
R10	67.16	55.51	12.91	43.1	116.7	21.15	52.76	37.37	1.72
R20	28.38	48.07	16.89	21.88	21.8	15.31	25.39	12.02	1.40
R40	2.679	7.821	2.855	2.182	10.88	2.988	4.90	3.59	0.69
P10	2.013	9.096	7.479	8.232	5.022	10.8	7.11	3.14	0.85
P20	4.366	1.801	5.495	2.555	2.454	2.244	3.15	1.45	0.50
R10C12	57.03	56.55	10.22	31.98	67.82	39.94	43.92	20.96	1.64
R20C12	28.42	13.54	**	**	23	14.91	19.97	7.01	1.30
R40C12	3.012	4.743	2.333	1.616	2.444	2.916	2.84	1.06	0.45
P10C12	4.377	6.783	6.92	2.923	6.878	2.502	5.06	2.06	0.70
P20C12	1.84	1.441	1.214	1.164	3.571	2.106	1.89	0.90	0.28
									#NUM!
									#NUM!
Implant	0.732	0.643	0.801	0.193	0.456	0.771	0.60	0.23	-0.22
Day 0	342.1	340	180.2	210.7	138.4	224.1	239.25	84.18	2.38

**** 31.15 (mix of R20CFZ12 3L & 1R)**

Week 2	1L	2L	3L	1R	2R	3R			
	HMG	HMG	HMG	HMG	HMG	HMG			Mean log10
UT	1000	576	870.7	635	933.6	1006	836.88	186.83	2.92
RS	0.728	3.537	2.549	1.562	3.317	3.987	2.61	1.26	0.42
RCLR	11.81	6.672	11.14	9.976	5.081	13.71	9.73	3.26	0.99
R10C25	10.59	11.96	6.61	13.79	9.694	18.84	11.91	4.15	1.08
R10	83.81	98.3	145.4	83.46	65.54	139.2	102.62	32.50	2.01
R20	4.817	4.145	8.189	2.775	4.813	9.6	5.72	2.61	0.76
R40	0.418	1.344	1.174	1.031	0.819	1.796	1.10	0.47	0.04
P10	0.522	0.473	0.237	0.361	0.26	0.221	0.35	0.13	-0.46
P20	0.971	2.386	0.703	0.864	0.504	0.397	0.97	0.73	-0.01
R10C12	15.57	6.252	9.439	14.73	19.04	11.07	12.68	4.64	1.10
R20C12	2.766	8.699	2.582	6.884	10.96	2.441	5.72	3.66	0.76
R40C12	1.207	0.198	0.136	1.522	0.24	0.19	0.58	0.62	-0.23
P10C12	0.924	1.015	0.717	0.776	0.248	1.035	0.79	0.29	-0.10
P20C12	0.146	0.236	0.82	0.139	0.307	0.593	0.37	0.27	-0.43

Week 4	1L	2L	3L	1R	2R	3R			
	**HMG**	**HMG**	**HMG**	**HMG**	**HMG**	**HMG**			**Mean log10**
UT	------	------	------	------	------	------	#DIV/0!	#DIV/0!	#DIV/0!
RS	0.022	0.046	0.034	0.12	0.117	0.052	0.07	0.04	-1.19
RCLR	1.582	0.251	0.036	0.578	0.046	0.068	0.43	0.60	-0.37
R10C25	0.018	0.054	0.362	0.048	0.108	0.057	0.11	0.13	-0.97
R10	2.22	13.45	------	0.389	3.072	------	4.78	5.89	0.68
R20	0.194	0.024	0.018	0.053	0.651	1.259	0.37	0.50	-0.44
R40	0.464	0.159	10.23	0.099	0.672	0.123	1.96	4.06	0.29
P10	0.028	0.138	0.621	0.569	0.101	0.538	0.33	0.27	-0.48
P20	0.062	0.035	0.076	0.111	0.053	0.088	0.07	0.03	-1.15
R10C12	0.088	0.396	0.211	0.62	0.376	0.068	0.29	0.21	-0.53
R20C12	0.034	0.043	0.081	0.676	0.132	0.047	0.17	0.25	-0.77
R40C12	0.059	0.11	0.231	0.039	0.026	0.053	0.09	0.08	-1.06
P10C12	0.043	0	0.21	0.213	0.003	0.105	0.10	0.10	-1.02
P20C12	0.116	0.026	0	0.029	0.06	0.204	0.07	0.08	-1.14
